# Integrative mechanisms and intervention targets of the microbiota–gut–brain axis in depressive disorders: advances across immune, endocrine, and central nervous system pathways

**DOI:** 10.3389/fpsyt.2026.1848918

**Published:** 2026-06-08

**Authors:** Hongyu Zhao, Limei Ao, Lingfang Hao, Yuxia Wei, Hong Zhen Yin, Xiao Qing Lee, Chenyu Guo, Zhenyi Wang, JinRui Yang, Ren Yang, Gai Lan Zhou

**Affiliations:** 1 Department of Gastroenterology, The Traditional Chinese and Mongolian Medicine Hospital of Hohhot, Huhhot, China; 2 College of Traditional Chinese Medicine, Inner Mongolia Medical University, Huhhot, China; 3 Department of Gastroenterology, Inner Mongolia Autonomous Region Traditional Chinese Medicine Hospital, Huhhot, China; 4 Shenzhen Longhua District People’s Hospital, Department of Emergency Medicine, Shenzhen, China; 5 College of Physical Education, Huaqiao University, Xiamen, China

**Keywords:** depressive disorders, gut microbiota, microbiota–gut–brain axis, neuroinflammation, psychobiotics, tryptophan–kynurenine pathway

## Abstract

Depressive disorders are highly heterogeneous syndromes characterized not only by depressed mood but also by cognitive impairment, sleep–circadian rhythm disturbances, altered appetite, somatic discomfort, and metabolic or gastrointestinal comorbidities. In recent years, the microbiota–gut–brain axis (MGBA) has been increasingly recognized as an integrative biological framework linking abnormalities in mood regulation, immune responses, endocrine function, metabolism, and neuroplasticity. This review provides a systematic synthesis of gut microbial ecology and host phenotypic features associated with depressive disorders, with particular emphasis on the depletion of short-chain fatty acid-producing commensals, the enrichment of potentially pro-inflammatory taxa, and the functional remodeling of key metabolic pathways, including the tryptophan–kynurenine pathway, short-chain fatty acids, bile acids, and trimethylamine N-oxide. We further discuss how bidirectional gut-to-brain and brain-to-gut communication may contribute to the onset and progression of depressive disorders through intestinal barrier disruption, low-grade systemic inflammation, hypothalamic–pituitary–adrenal axis activation, vagal signaling, and dysregulation of neurotransmitter and neurotrophic pathways. Current interventional evidence suggests that dietary and lifestyle modification, psychobiotics, and fecal microbiota transplantation may exert antidepressant potential in selected populations; however, the overall effect sizes remain limited and between-study heterogeneity is substantial. Patients with prominent gastrointestinal symptoms, metabolic abnormalities, or low-grade inflammatory states may represent priority candidates for MGBA-targeted interventions; nevertheless, a putative microbiota-responsive phenotype should not be simply equated with high stress exposure alone, and its definition requires prospective validation integrating stress burden, host responses, and microbial/metabolic readouts. Overall, MGBA research is gradually moving beyond descriptive profiling of microbial composition toward functional integration and clinical translation; however, causal inference, multi-omics standardization, and the identification of stratification biomarkers remain major challenges. Future studies should incorporate phenotype-based stratification, strengthened functional readouts, and precision intervention designs to determine which patients are most likely to benefit from microbiota-targeted therapies.

## 1 Introduction

Depressive disorders are among the leading contributors to the global burden of mental disorders, characterized by high prevalence, frequent recurrence, and substantial disability, and are closely associated with increased suicide risk and considerable socioeconomic burden (1). Notably, depressive disorders should not be regarded as a single, homogeneous disease entity, but rather as highly heterogeneous clinical syndromes. Beyond persistent low mood and anhedonia, affected individuals may present with cognitive impairment, sleep–circadian rhythm disturbances, changes in appetite and body weight, fatigue, pain, and a range of gastrointestinal or somatic symptoms, including abdominal bloating, constipation, and diarrhea. At the comorbidity level, depressive disorders are often intertwined with anxiety disorders, substance use disorders, metabolic syndrome, obesity, chronic inflammatory diseases, and irritable bowel syndrome (2). Substantial interindividual differences in symptom profiles, comorbidity burden, illness trajectories, and treatment responses suggest that the traditional explanatory framework centered on a single central neurotransmitter imbalance is no longer sufficient to capture the complex biological underpinnings and clinical heterogeneity of depressive disorders.

In recent years, the microbiota–gut–brain axis (MGBA) has emerged as an important integrative framework in research on depressive disorders. This framework emphasizes that the gut microbial ecosystem, intestinal mucosal barrier, mucosal and systemic immunity, neuroendocrine regulation, and central neural networks do not operate in isolation, but are engaged in continuous bidirectional communication and dynamic feedback (3). From this perspective, depressive disorders should not be viewed solely as consequences of dysregulated neural circuits involved in emotional regulation, but may also be associated with gut microbial dysbiosis, low-grade inflammation, maladaptive stress responses, metabolic pathway remodeling, and somatic comorbidity backgrounds. Therefore, the significance of the MGBA does not lie in replacing existing central nervous system theories with a single gut-derived hypothesis, but rather in providing a more systematic explanatory framework for understanding the psychosomatic comorbidity features, biological heterogeneity, and potential stratified intervention strategies of depressive disorders (4).

Current preclinical and clinical evidence suggests a close association between depressive disorders and alterations in the composition and functional capacity of the gut microbiota. Evidence from animal models indicates that microbiota depletion, microbial perturbation, or transplantation of microbiota derived from depressed donors can influence stress responses, emotion-related behaviors, and neuroendocrine status in recipient animals. In human studies, patients with depressive disorders frequently exhibit gut microbial dysbiosis, abnormalities in microbial metabolic pathways, and host phenotypic alterations associated with inflammation, metabolism, and gastrointestinal symptoms (5, 6). However, the current evidence remains subject to several important limitations. First, many studies are still largely confined to the descriptive characterization of differences in microbial composition, with insufficient integration of immune, endocrine, neural, and metabolic pathways. Second, the correspondence between distinct clinical subtypes of depressive disorders and microbial ecological features remains unclear, making it difficult to address the clinically critical question of which patients are most likely to benefit from microbiota-targeted interventions. Third, existing studies continue to face substantial challenges in causal inference, multi-omics standardization, control of confounding factors, and the identification of translational biomarkers. These limitations indicate that, for MGBA research to genuinely inform stratified diagnosis and treatment of depressive disorders, the field must move beyond correlative descriptions toward mechanistic integration and clinical translation (7–9). Based on these considerations, this review aims to comprehensively synthesize research on MGBA and depressive disorders from the perspectives of mechanistic integration and clinical translation. Particular attention is given to depression-related alterations in the gut microbial ecosystem and host phenotypic features, with a focus on the roles of immune-inflammatory processes, stress-related neuroendocrine regulation, neurotransmitter systems, and key metabolic networks in bidirectional gut–brain communication. Potential monitoring indicators, microbiota-targeted intervention strategies, and clinically relevant translational directions are also discussed. This article was designed as a narrative review. The literature was mainly retrieved from PubMed, Web of Science, Embase, and related databases, with emphasis on recent preclinical studies, clinical observational studies, randomized controlled trials, and representative reviews concerning gut microbial alterations, key metabolic pathways, immune-inflammatory mechanisms, bidirectional brain–gut communication, and microbiota-targeted interventions in depressive disorders. This review primarily summarizes and discusses existing evidence in this field through the lens of mechanistic integration and clinical translation.

## 2 Gut microbial ecology and host phenotypes associated with depressive disorders

### 2.1 Alterations in microbial diversity and composition

Population-based studies using 16S rRNA sequencing and metagenomic approaches generally suggest that depressive disorders are closely associated with disturbances in gut microbial diversity and community structure; however, findings regarding specific α-diversity metrics remain inconsistent (10). Several early case–control studies reported that, compared with healthy controls, patients with major depressive disorder (MDD) exhibited reduced gut microbial α-diversity and marked separation in β-diversity, suggesting a reconfiguration of the overall microbial ecological landscape (11). However, systematic reviews and meta-analyses integrating multiple studies have shown that, across diverse populations and analytical platforms, differences in α-diversity are often inconsistent. Reductions in richness indices such as Chao1 have been observed only in certain subgroups, such as antidepressant-free Western participants, highlighting the substantial influence of dietary patterns, medication exposure, and methodological approaches on microbial diversity metrics (12). Multiple case–control studies and systematic reviews have consistently reported a depletion of anti-inflammatory, butyrate-producing commensal genera in patients with depressive disorders, accompanied by the relative enrichment of several genera associated with inflammatory responses and mucin degradation. Typical findings include reduced abundances of short-chain fatty acid-producing genera, such as *Faecalibacterium*, *Coprococcus*, and *Butyricicoccus*, with decreases in *Faecalibacterium* showing a negative association with depression severity across multiple cohorts. Conversely, *Eggerthella*, *Flavonifractor*, Enterobacteriaceae, and certain *Streptococcus* and *Alistipes* taxa, which are considered potentially pro-inflammatory or opportunistic pathobionts, tend to be enriched in depressive populations (13).

Large-scale cohort-based metagenomic studies have further strengthened this view at a higher taxonomic and functional resolution. Analyses based on population cohorts such as the Flemish Gut Flora Project and LifeLines DEEP showed that the relative abundances of several butyrate-producing genera were not only reduced in individuals with depression or higher depression scores, but also positively associated with quality-of-life scores and negatively associated with depressive symptoms. These studies also highlighted the importance of confounding factors, including dietary patterns, body mass index, and psychotropic medication use, in interpreting associations between the gut microbiota and psychiatric phenotypes (14). Consistent with findings from umbrella meta-analyses across psychiatric populations, depressive disorders may not be characterized by a single, stable, and depression-specific microbial signature at the level of gut microbial ecology. Instead, they appear to reflect a shared dysbiotic pattern characterized by the depletion of butyrate-producing bacteria and the enrichment of pro-inflammatory taxa, shifting toward a state of low-grade inflammation and impaired barrier function, with several variants emerging under different host backgrounds and environmental exposures (15). It should be noted that the evidence for the above-mentioned differences in microbial composition is still derived primarily from cross-sectional and case–control studies, and the findings are susceptible to the influence of dietary patterns, geographic and population characteristics, medication exposure, sample size, and the extent to which psychiatric comorbidities are controlled. Therefore, at the current stage, these findings are better interpreted as signals of depression-related community dysbiosis, rather than as stable microbial signatures that can be directly applied to stratified diagnosis.

### 2.2 Functional and metabolic alterations: insights from metagenomic and metabolomic perspectives

Compared with the substantial between-study heterogeneity observed in the taxonomic composition of the gut microbiota, abnormalities in the functional and metabolic pathways of the gut microbial ecosystem associated with depressive disorders appear to be more consistent and may be more closely linked to potential mechanistic hubs and clinically translatable endpoints (12). Current metagenomic and targeted or untargeted metabolomic studies generally indicate that microbial functional remodeling in individuals with depression is mainly concentrated in the tryptophan–kynurenine pathway, short-chain fatty acid production, bile acid transformation, and other key pathways related to nitrogen metabolism, energy metabolism, and cofactor biosynthesis. These alterations are closely associated with inflammatory burden, hypothalamic–pituitary–adrenal (HPA) axis activity, affective–cognitive phenotypes, and certain somatic comorbidities (15–19). Compared with the increase or decrease of a specific bacterial genus, these functional readouts are more likely to exhibit directionally consistent abnormal patterns across different cohorts. This suggests that the core of depression-related microbial dysbiosis may not lie in the presence of a single, stable, and depression-specific microbial signature, but rather in the convergence of diverse microbial configurations onto several relatively convergent abnormalities in metabolic and signaling pathways.

From a research perspective, abnormalities at the functional and metabolic levels deserve particular attention not only because they facilitate the integration of immune-inflammatory processes, stress-related neuroendocrine regulation, and central neural plasticity, but also because they are more suitable as candidate readouts for monitoring, stratification, and intervention. Particularly in the context of the marked clinical heterogeneity of depressive disorders and the recurrent inconsistency of findings on microbial composition, constructing a microbial ecology–host response framework around key metabolic hubs may be more informative than merely cataloguing microbial alterations for explaining interindividual differences in symptom profiles, comorbidity burden, and treatment responses. **Table 1** summarizes the key metabolic pathways repeatedly reported in depressive disorders and their major host-related effects.

**Table 1:** Overview of key gut microbiota-related metabolites and host effects in depressive disorders.

Metabolic pathway/key metabolites	Typical alterations in depressive disorders (trend)	Major host effects/related axes	Main clinical associations	References
Short-chain fatty acids (SCFAs: acetate, propionate, butyrate, etc.)	Fecal and/or circulating SCFAs are generally decreased, particularly butyrate and propionate; however, subtype-specific changes vary across sample types, metabolic backgrounds, and exposure contexts.	Maintain intestinal epithelial barrier integrity; modulate Treg/Th17 balance and microglial activation via GPR41/43 signaling and HDAC inhibition; influence energy metabolism and neuroplasticity.	Associated with depression severity, inflammatory markers, and treatment outcomes, with interpretation influenced by diet, BMI, metabolic status, and medication exposure.	(20, 21)
Tryptophan–kynurenine (TRP–KYN) pathway	Decreased plasma/serum TRP, increased KYN/TRP ratio, elevated neurotoxic metabolites such as QUIN and 3-HK, and a KYNA/QUIN imbalance toward neurotoxicity.	Redirects tryptophan metabolism from 5-HT synthesis toward the KYN pathway; downstream KYN remodeling affects neuroplasticity through NMDA receptor signaling, oxidative stress, and neuroinflammation.	Associated with depressive symptoms, poor treatment response, cognitive impairment, and suicide risk.	(22, 23)
Indole and its derivatives (IPA, IAA, ILA, etc.)	Indole derivatives, especially IPA, are often reduced and are related to indole-producing bacteria such as *Clostridium* and *Bacteroides*.	Modulate antioxidant, anti-inflammatory, and barrier-protective responses mainly through aryl hydrocarbon receptor (AhR) signaling.	Associated with depressive symptoms, quality of life, and cognitive impairment.	(14, 24)
Bile acid profile (primary and secondary bile acids and related pathways)	Bile acid profiles are remodeled, with specific secondary bile acids linked to depressive symptoms and cognitive impairment, while the proportion of primary bile acids tends to decrease.	Regulate glucose and lipid metabolism, inflammation, HPA axis activity, and brain function through FXR/TGR5 signaling.	Associated with depressive symptoms, cognitive function, and metabolic symptoms.	(18, 25)
Trimethylamine N-oxide (TMAO)	Serum TMAO levels are increased and associated with depressive symptoms, metabolic syndrome, and elevated cardiovascular event risk.	Reflects the diet–microbiota–choline/carnitine metabolism axis and may promote inflammation, oxidative stress, and endothelial dysfunction.	Significantly associated with depressive symptoms, cardiovascular comorbidities, and metabolic risk.	(18)
B vitamins/Folate-related metabolism	Low folate and vitamin B12 levels, together with elevated homocysteine, are associated with increased risk of depressive disorders.	Regulate neurotransmitter synthesis through one-carbon metabolism and methylation-related pathways.	Folate, vitamin B12, and homocysteine are associated with depressive symptoms; L-methylfolate may enhance antidepressant efficacy.	(26)

### 2.3 Psychosomatic comorbidity and the “microbial ecological phenotype”

In real-world populations, depressive disorders frequently coexist with metabolic diseases, such as metabolic syndrome, obesity, and type 2 diabetes, as well as irritable bowel syndrome (IBS) and functional gastrointestinal disorders (FGIDs). These comorbid conditions may themselves influence the gut microbial ecosystem through chronic low-grade inflammation, insulin resistance, and altered visceral sensitivity (27). In patients with depressive disorders accompanied by metabolic abnormalities, the gut microbiota and metabolic profiles are often jointly shaped by both the depressive state and the background of metabolic comorbidity; therefore, these abnormal patterns should not be simply interpreted as depression-specific alterations. Existing studies suggest that individuals with MDD and overweight/obesity may exhibit enrichment of fatty acid metabolism-related genera, such as *Succinivibrio* and *Megamonas*, along with a reduction in potentially beneficial taxa such as *Bifidobacterium*, accompanied by systematic remodeling of lipid and bile acid pathways. However, it should be noted that these changes partially overlap with microbial alterations observed in individuals with obesity or metabolic syndrome alone; therefore, they are better regarded as risk-enhancing signals in the context of depression with metabolic dysregulation, rather than as established depression-specific microbial markers (28). With respect to gastrointestinal comorbidity, IBS frequently coexists with anxiety and depressive symptoms. Fecal 16S rRNA sequencing and metagenomic studies have shown that patients with diarrhea-predominant IBS (IBS-D) and those with depression share similar microbial signatures, including reduced α-diversity, enrichment of certain opportunistic pathobionts, and depletion of short-chain fatty acid-producing bacteria (29). Integrated fecal microbiome–metabolome multi-omics analyses further indicate that IBS patients with prominent psychological symptoms or high stress levels also exhibit distinct metabolic patterns involving tryptophan/5-HT metabolism and lipid metabolism-related metabolites (30).

Taken together, studies of populations with metabolic and gastrointestinal comorbidities suggest that patients with depressive disorders differ in their metabolic–inflammatory burden, gastrointestinal symptom profiles, and corresponding microbial and metabolic features, with some subgroups showing relatively stable combinatorial patterns. The correspondence of these patterns with immune and endocrine activation pathways and clinical presentations, such as affective symptom-dominant or somatic/gastrointestinal symptom-dominant profiles, suggests the existence of candidate subtypes of depressive disorders defined by microbial ecological and metabolic characteristics. However, these candidate subtypes should not necessarily be equated with depression-specific biological stratification, as they may also partly reflect host–microbiota co-shifts driven by metabolic comorbidity. Therefore, whether these subtypes reflect upstream etiological factors, downstream stress- or metabolism-related manifestations, or the combined effects of multiple comorbid conditions remains to be determined through longitudinal follow-up and interventional studies.

## 3 Systemic framework and signaling basis of MGBA

### 3.1 Structural and cellular basis of MGBA

MGBA is essentially a hierarchical network composed of multiple anatomical structures and cellular–tissue units. At its peripheral end, the MGBA comprises the extensive microbial communities within the intestinal lumen, the mucus layer covering the epithelial surface, and the single-layer intestinal epithelial barrier whose integrity is maintained by tight junctions. Beneath this barrier, the lamina propria contains abundant gut-associated lymphoid tissue (GALT), dendritic cells, macrophages, and various T- and B-cell subsets, which together constitute the first line of mucosal immune defense (31). At the neural level, the enteric nervous system (ENS) embedded within the intestinal wall finely regulates intestinal motility, secretion, and local blood flow through the myenteric and submucosal plexuses. It can function as a local integrative system often referred to as the “second brain”, while also engaging in bidirectional information exchange with the central nervous system (CNS) via the vagus nerve and sympathetic pathways (32). The autonomic nervous system, including its sympathetic and parasympathetic branches, together with the hypothalamic–pituitary–adrenal (HPA) axis, constitutes the major effector pathways of the stress response and represents a core component of the central arm of the MGBA.

In addition, enteroendocrine cells scattered throughout the intestinal epithelium, including 5-HT-secreting enterochromaffin cells and L cells that secrete GLP-1, PYY, and other peptides, act as sensory–effector transducers within the MGBA. These cells detect changes in luminal nutrients and microbial metabolites and transmit signals to the brainstem and higher brain centers through hormone secretion and synapse-like contacts with vagal nerve terminals. Conversely, neural and hormonal signals from the central nervous system can regulate intestinal function and the local microenvironment through these cells (33).

### 3.2 Molecular signals and metabolic mediators of MGBA

On the basis of the structural framework described above, information transfer within MGBA relies primarily on the coordinated actions of multiple molecular signals and metabolic mediators. These signals can be broadly categorized into three interconnected levels: immune signaling, metabolic signaling, and neuroendocrine signaling (34). These three categories do not operate in isolation; rather, they form a continuous communication network spanning the intestinal mucosal barrier, local microenvironment, peripheral circulation, and central nervous system. Together, they determine how intestinal signals are sensed, amplified, and integrated by the host into responses at the levels of brain function and behavior. Among these, immune signaling mainly arises from interactions between the gut microbiota and its associated molecules and the host mucosal immune system. Microbe-associated molecular patterns and the inflammatory mediators they induce not only participate in maintaining local intestinal immune homeostasis, but may also, under specific conditions, promote the spread of inflammatory signals from the gut to the systemic compartment, thereby serving as important mediators linking peripheral microbial ecological alterations to systemic host responses (35, 36). Metabolic signaling mainly reflects the ability of the gut microbiota to remodel nutritional substrates and host metabolic networks, involving short-chain fatty acids, bile acids, tryptophan-related metabolites, and other molecules related to energy metabolism and one-carbon metabolism. These mediators not only reflect the functional status of the microbiota, but also serve as important bridges linking microbial activity to intestinal barrier homeostasis, immune tone, and the regulation of brain function (18, 35).

**Figure 1:**
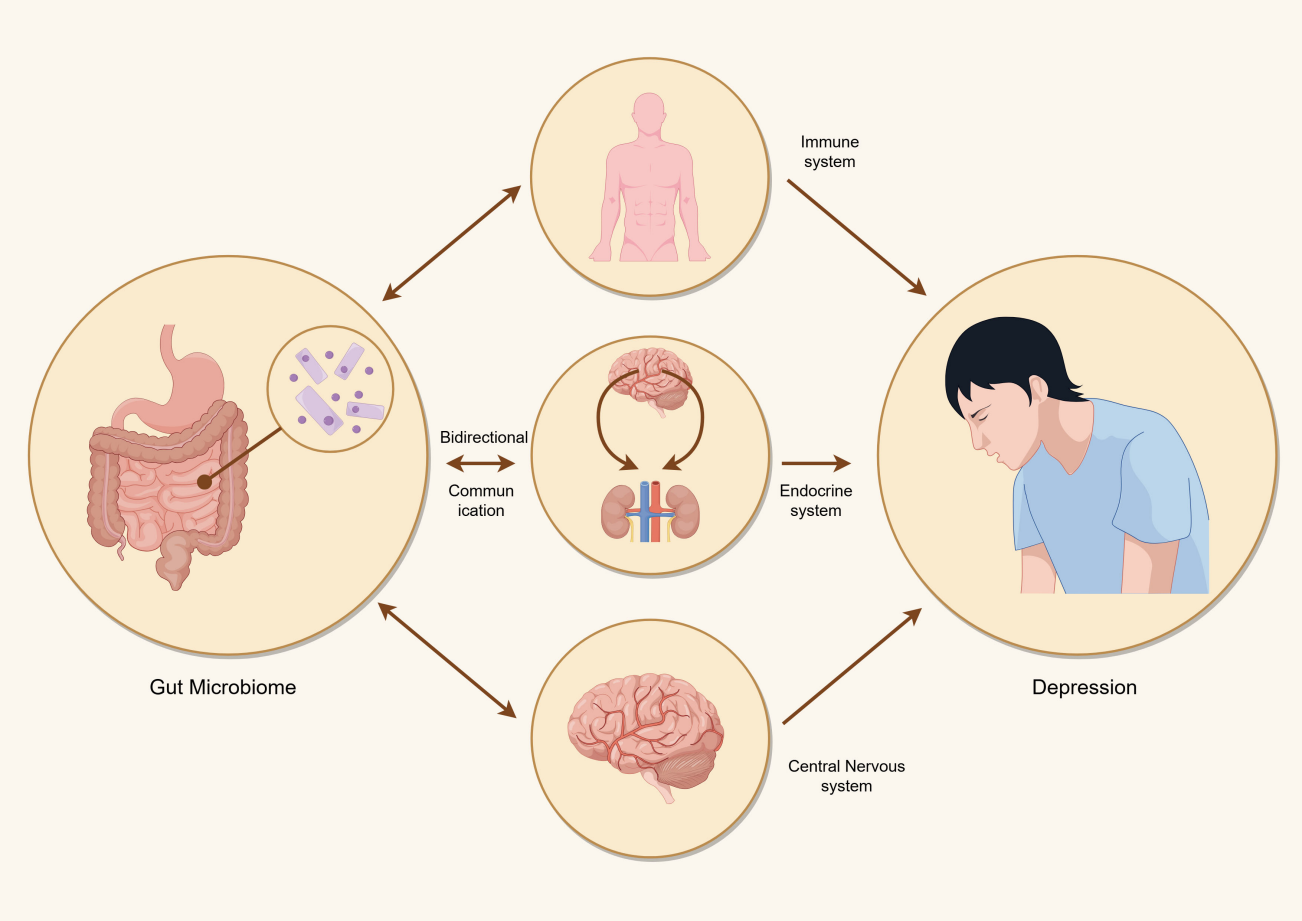
Overall framework of MGBA dysregulation in depressive disorders. This figure summarizes the bidirectional relationships among gut microbial dysbiosis, intestinal barrier disruption, immune-inflammatory activation, key metabolic abnormalities, HPA axis hyperresponsiveness, and impaired neuroplasticity, and highlights that gut-to-brain and brain-to-gut signaling jointly contribute to the onset and maintenance of depressive disorders through continuous interactions. Arrows indicate the principal direction of action, whereas bidirectional arrows indicate reciprocal interactions between processes.

Neuroendocrine information transfer is closely intertwined with the two categories of signaling described above. Enteroendocrine cells, the enteric nervous system, autonomic neural pathways, and the HPA axis together constitute key effector systems within the MGBA, mediating gut-to-brain signaling and subsequent central-to-gut feedback regulation. Neurotransmitters and their precursors, gut-derived hormones, and stress-related hormones do not act in isolation; rather, they form dynamic coupling among local sensory apparatuses, vagal afferent signaling, autonomic efferent output, and neuroendocrine regulation, thereby transforming microbial signals within the intestinal lumen into higher-order physiological and behavioral responses (37). Taken together, the key feature of the MGBA does not lie in the increase or decrease of any single signaling molecule, but rather in the dysregulated coupling among immune, metabolic, and neuroendocrine networks. **Figure 1** provides a schematic overview of the overall dysregulation framework of the MGBA in depressive disorders, facilitating a systems-level understanding of the interactions among intestinal barrier disruption, immune-inflammatory activation, key metabolic abnormalities, HPA axis hyperresponsiveness, and impaired neuroplasticity. Based on the structural foundations and molecular mediators described above, MGBA abnormalities associated with depressive disorders can be broadly categorized into two interrelated pathways: first, a gut-to-brain ascending pathway, in which local intestinal disturbances are transmitted to the central nervous system through immune-inflammatory and neural signaling; and second, a brain-to-gut descending pathway, in which chronic stress and brain network abnormalities reshape the intestinal microenvironment through autonomic and neuroendocrine regulation. These two pathways do not operate in isolation, but jointly contribute to the onset and maintenance of depressive disorders through continuous reciprocal interactions.

## 4 Gut-to-brain ascending pathways: inflammatory and neural routes

### 4.1 Intestinal barrier disruption and “leaky gut”

The intestinal barrier is maintained in part by tight junction proteins, such as ZO-1 and occludin, which regulate intestinal permeability and prevent the translocation of harmful luminal substances. The “leaky gut” phenomenon refers to increased intestinal epithelial permeability and reduced expression of tight junction proteins, allowing microbial components and metabolites from the intestinal lumen to enter the systemic circulation and trigger low-grade systemic inflammation (38). Lipopolysaccharide (LPS), a prototypical endotoxin derived from gut Gram-negative bacteria, can enter the systemic circulation through the portal venous system under conditions of increased intestinal permeability, thereby activating immune responses and further exacerbating central nervous system inflammation (39). Increased intestinal permeability, often referred to as “leaky gut,” appears to be relatively common in patients with depression. Studies have shown that patients with depression exhibit elevated plasma levels of LPS, intestinal fatty acid-binding protein (I-FABP), and soluble CD14 (sCD14), suggesting intestinal barrier disruption and the translocation of microbial components into the bloodstream (40). LPS can activate the peripheral immune system and exacerbate neuroinflammation, ultimately affecting mood and cognitive function. Meanwhile, leaky gut may further promote immune–neural axis dysregulation and increase the risk of neuroinflammation by altering the levels of metabolites such as short-chain fatty acids (SCFAs) (41).

### 4.2 Peripheral inflammation, the HPA axis, and the blood–brain barrier

After endotoxins such as LPS translocate into the bloodstream through increased intestinal permeability, they can activate peripheral immune cells and induce the release of cytokines, including IL-6 and TNF-α. These inflammatory signals may affect the brain through blood–brain barrier (BBB)-dependent and BBB-independent routes, thereby promoting neuroinflammation. Pro-inflammatory cytokines can activate the hypothalamic–pituitary–adrenal (HPA) axis, leading to excessive glucocorticoid secretion and, under chronic stress conditions, glucocorticoid resistance or impaired negative feedback. This process may sustain immune activation and contribute to an inflammation–stress positive feedback loop (42). Excessive glucocorticoid secretion may, in turn, aggravate intestinal barrier disruption, further facilitating the translocation of harmful luminal substances such as LPS and thereby forming a vicious cycle between stress activation, barrier impairment, and inflammatory signaling (43). Activation of the HPA axis exerts important effects on the BBB. Studies suggest that glucocorticoids can modulate BBB permeability by affecting endothelial transport pathways and barrier-regulating structures, thereby facilitating the entry or signaling of inflammatory mediators across the BBB and exacerbating central immune responses (44).

### 4.3 Central immune activation and impaired neuroplasticity

Peripheral inflammatory signals can affect brain-resident immune cells, such as microglia, through BBB-dependent mechanisms, thereby activating neuroinflammatory responses. Microglia are the principal immune cells of the brain and can sense peripheral immune signals while regulating the neural microenvironment. Neuroinflammation may aggravate depressive symptoms by impairing neuroplasticity, disrupting synaptic connectivity, and inhibiting neuronal growth (45). In specific brain regions, such as the prefrontal cortex (PFC), hippocampus, and amygdala, neuroinflammation and structural and functional alterations are closely associated with depressive symptoms (46). In patients with depressive disorders, these brain regions often show reduced gray matter density and abnormal functional activity, which are significantly associated with peripheral immune responses and gut microbial dysbiosis (47). In this process, reduced neuroplasticity is considered a key factor affecting emotional regulation and cognitive function.

### 4.4 Sensing and transmission of intestinal states by the enteric nervous system and the vagus nerve

In addition to inflammation-mediated ascending pathways, intestinal signals can also influence central function through the ENS, the vagus nerve, and neurotransmitter-related mechanisms, thereby constituting another important route of gut-to-brain communication in depressive disorders. The ENS consists of dense networks of neurons and glial cells distributed within the gastrointestinal wall and can regulate intestinal motility, secretion, and local blood flow to a considerable extent independently of the CNS (48). The ENS is extensively connected to the central nervous system through the vagus nerve and spinal afferent fibers. As the major parasympathetic pathway, the vagus nerve rapidly transmits mechanical, chemical, and inflammatory signals from the intestinal lumen to the nucleus tractus solitarius in the medulla and higher brain regions, thereby participating in the regulation of stress responses, emotion, and homeostasis (49). Recent morphological and functional studies further suggest that a subset of enteroendocrine cells can form neuropod-like structures and establish synapse-like contacts with vagal nerve terminals, thereby translating luminal nutrients, microbe-associated molecules, and their metabolites into neuroelectrochemical signals that modulate vagal afferent firing patterns and ENS activity (50). This suggests that intestinal sensing of external and internal environmental changes does not rely solely on slow humoral pathways, but can also dynamically regulate brain function and behavioral states through rapid neural transmission.

The role of the vagus nerve in the ascending transmission of microbial signals has been supported relatively directly by preclinical studies. A classic animal study showed that chronic oral administration of *Lactobacillus rhamnosus* JB-1 altered the expression of GABA receptor subunits in the mouse brain and reduced anxiety- and depression-like behaviors. However, these behavioral and neurochemical effects were abolished after subdiaphragmatic vagotomy, indicating a clear vagus nerve-dependent mechanism by which specific bacterial strains modulate emotional behavior (51, 52). Further electrophysiological studies have also shown that JB-1 can rapidly increase the firing frequency of mesenteric vagal afferent fibers, providing direct evidence for a fast communication route along the luminal signal–vagus nerve–brain pathway (53).

By contrast, current human studies provide mainly indirect supportive evidence. Some studies have observed improvements in heart rate variability indices after probiotic or multi-strain psychobiotic interventions, accompanied by partial alleviation of depressive symptoms or improvement in sleep quality, suggesting that vagus nerve-related measures may be involved in host responses to microbiota-based interventions (54). However, it should be noted that measures such as heart rate variability cannot be directly equated with the vagus nerve dependence demonstrated in animal experiments, and current evidence is still insufficient to prove the existence of a vagus nerve-dependent mechanism in human depressive disorders at the same level as that observed in the JB-1 animal model. Therefore, the vagus nerve is better regarded as a biologically plausible neural interface within the MGBA, whereas its specific effect size, target populations, and clinical translational relevance in human depressive disorders remain to be clarified through more rigorous mechanistic studies.

### 4.5 Neurotransmitters and neuroplasticity

Within the MGBA, neurotransmitters and the metabolism of their precursors constitute an important molecular bridge linking intestinal signals to changes in brain function. Among these, the 5-hydroxytryptamine (5-HT) system is one of the most extensively studied pathways. More than 90% of peripheral 5-HT is synthesized by enterochromaffin cells in the intestinal mucosa under the catalysis of tryptophan hydroxylase 1 (TPH1). This gut-derived 5-HT participates in the regulation of intestinal motility and visceral sensation and may indirectly influence the central 5-HT system and emotion-related brain regions through vagal and humoral pathways (55). Animal and *in vitro* studies have shown that microbial metabolites, such as short-chain fatty acids, can upregulate TPH1 expression in enterochromaffin cells and promote colonic 5-HT production, suggesting that specific gut microbes and their metabolites may influence host emotional states by regulating tryptophan utilization and 5-HT availability (56, 57). Meanwhile, a shift in tryptophan metabolism from the 5-HT synthetic branch toward the kynurenine (KYN) pathway has been repeatedly reported in cohorts of patients with depressive disorders and in animal models, characterized by reduced peripheral TRP levels, an increased KYN/TRP ratio, and the accumulation of neurotoxic metabolites. The gut microbiota may participate in this critical metabolic diversion by modulating tryptophan availability, local inflammation, and IDO/TDO activity (58, 59).

Beyond 5-HT, gut microbes may also participate in the regulation of pathways related to amino acid neurotransmitters, including γ-aminobutyric acid (GABA) and glutamate (60). Various *Lactobacillus* and *Bifidobacterium* species possess the potential to produce GABA, and some preclinical studies suggest that strain-mediated regulation of GABA signaling and emotional behavior may be vagus nerve-dependent. However, the translational relevance of this mechanism in human depressive disorders remains to be further validated (60, 61). Therefore, in research on the depression-related MGBA, the key issue is not simply how much bioactive neurotransmitter is directly produced by bacteria, but rather how the microbiota and its metabolites indirectly shape the functional state of central neurotransmitter networks by regulating neurotransmitter precursor availability, receptor expression, local neural transmission, and the inflammatory milieu.

Brain-derived neurotrophic factor (BDNF) represents another key node linking alterations in the gut microbial ecosystem to abnormalities in neuroplasticity. Animal studies have shown that SCFAs, particularly butyrate, can improve depression-like behaviors by regulating histone acetylation, upregulating hippocampal BDNF/cAMP response element-binding protein (CREB) signaling, and suppressing neuroinflammation (62). In human studies, several randomized controlled trials and subsequent meta-analyses have suggested that specific psychobiotic interventions, particularly multi-strain formulations containing *Lactobacillus* and *Bifidobacterium*, may modestly reduce depression scores. A limited number of mechanistic studies have also observed increased serum BDNF levels or reduced KYN/TRP ratios, providing preliminary clinical support for the hypothesis that the microbiota may influence depressive symptoms by modulating neurotransmitter–neuroplasticity pathways (63, 64). Overall, 5-HT/TRP–KYN metabolism, GABA-related signaling, and the SCFA–BDNF axis together constitute representative networks involved in neurotransmitter and neuroplasticity regulation in depressive disorders, providing biological clues for understanding therapeutic heterogeneity and potential stratified intervention strategies from the perspective of the MGBA.

### 4.6 Immune–neural–endocrine crosstalk in ascending pathways

**Figure 2:**
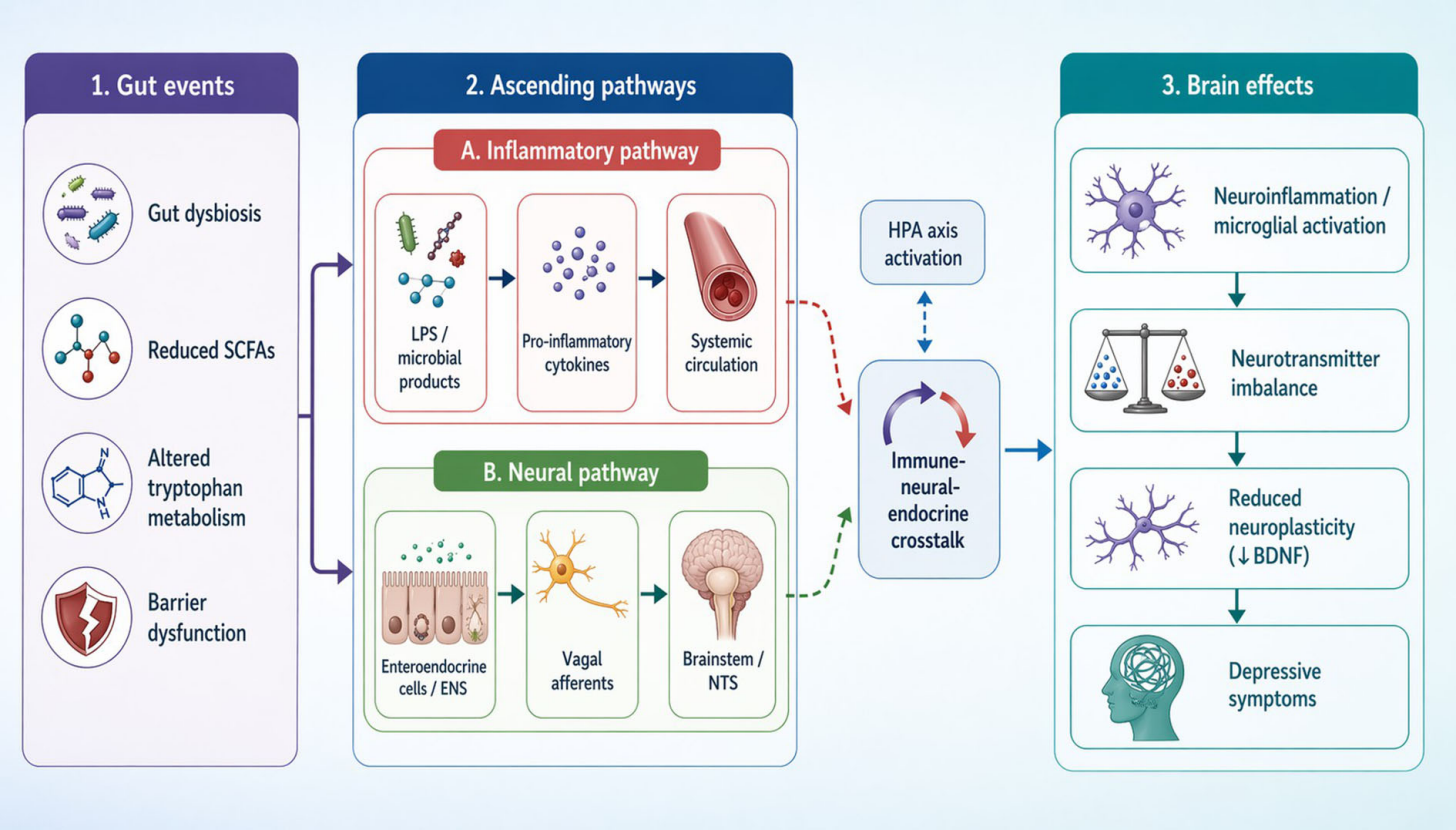
Integrated framework of gut-to-brain ascending pathways in depressive disorders. Starting from gut microbial dysbiosis, reduced short-chain fatty acids, abnormal tryptophan metabolism, and impaired intestinal barrier function, this figure summarizes the major processes by which inflammatory and neural pathways transmit signals to the central nervous system. The inflammatory pathway mainly involves LPS/microbe-associated products, pro-inflammatory cytokines, and systemic circulating signals, whereas the neural pathway primarily involves enteroendocrine cells/the ENS, vagal afferent signaling, and brainstem integration. Under the influence of HPA axis activation and immune–neural–endocrine crosstalk, these two pathways jointly affect neuroinflammation, neurotransmitter homeostasis, and neuroplasticity, thereby contributing to the development of depressive symptoms. Arrows indicate the principal direction of action.

From the perspective of ascending pathways as a whole, immune-inflammatory processes, vagal signaling, and endocrine regulation do not operate independently; instead, they continuously intersect and amplify one another during the transmission of local intestinal abnormalities to the central nervous system, together forming an integrated network of gut-to-brain communication in depressive disorders. Intestinal barrier disruption and microbial metabolic abnormalities can alter peripheral inflammatory tone and influence central responses through vagal afferent signaling and brainstem integration. Meanwhile, stress-related endocrine states may further modulate inflammatory signaling and neurotransmitter-related pathways, thereby strengthening the effects of gut-derived abnormalities on brain function (65, 66). Therefore, gut-to-brain ascending abnormalities in depressive disorders are better understood as a network-level dysregulation involving multiple systems, rather than as several isolated single-mechanism chains. Interpreting immune, neural, and endocrine signals within a unified framework not only helps explain interindividual differences in symptom spectra and phenotypes, but also provides a logical basis for understanding brain-to-gut descending remodeling from the perspective of bidirectional communication. **Figure 2** summarizes this gut-to-brain ascending regulatory framework in depressive disorders.

## 5 Brain-to-gut descending pathways: stress, brain networks, and remodeling of the intestinal microenvironment

In MGBA dysregulation associated with depressive disorders, the brain-to-gut descending pathway is not a secondary component, but rather an important process intertwined with gut-to-brain ascending signaling. Chronic stress and abnormalities in brain networks can continuously influence intestinal motility, secretion, mucosal immunity, and barrier homeostasis through autonomic and neuroendocrine regulation, thereby reshaping the intestinal microenvironment and contributing to the maintenance of microbial dysbiosis and maladaptive host responses.

### 5.1 Chronic stress, the HPA axis, and intestinal function

Chronic stress-induced abnormalities in brain-to-gut descending regulation are first manifested as sustained activation of the HPA axis. Persistent psychological or physiological stress can promote the release of corticotropin-releasing hormone (CRH) from the hypothalamic paraventricular nucleus, subsequently driving pituitary secretion of adrenocorticotropic hormone (ACTH) and ultimately increasing adrenal glucocorticoid secretion (67). In depressive disorders and stress-related phenotypes, this axis often exhibits increased basal activity, impaired negative feedback regulation, and circadian rhythm disruption (68). This hyperresponsive state of the HPA axis is not only an important marker of central stress responses, but also a key upstream driver of descending remodeling of the intestinal microenvironment.

At the gastrointestinal level, HPA axis activation and concomitant sympathetic excitation can jointly alter intestinal motility, secretion, and mucosal blood flow, thereby affecting the stability of the intestinal luminal ecological niche (69). Glucocorticoid and catecholamine signaling can also act on intestinal epithelial cells, the ENS, and mucosal immune cells, inducing disruption of tight junction homeostasis, weakening of mucus layer defense, and shifts in local immune responses, ultimately leading to increased intestinal permeability and impaired barrier function (70). It should be emphasized that chronic stress does not affect the intestine solely through direct hormonal actions; rather, it continuously reshapes the intestinal motility, secretory, defensive, and metabolic milieu through a coupled HPA axis–autonomic nervous system–mucosal immunity–epithelial barrier network (71, 72). Therefore, from the perspective of the brain-to-gut descending pathway, the central significance of chronic stress lies in its ability to chronically shape an intestinal internal environment that is more prone to microbial dysbiosis and functional disturbance, rather than merely inducing transient intestinal responses.

### 5.2 Depression-related brain networks and autonomic regulation

Brain network abnormalities associated with depressive disorders are not limited to emotional processing, but also involve the higher-order integration of interoception, autonomic output, and visceral homeostatic regulation. Structures such as the prefrontal cortex, anterior cingulate cortex, insula, amygdala, and hypothalamus constitute key nodes of the central autonomic network (CAN) (73). Among these structures, the insula–anterior cingulate system plays a hub-like role in salience detection and interoceptive prediction. Under stress conditions, this network can synchronously drive autonomic and neuroendocrine responses; therefore, its functional deviation may constitute an important central basis for brain-to-gut descending signaling (74). In the context of depressive disorders, such network abnormalities are commonly manifested as sympathetic–parasympathetic imbalance and corresponding alterations in visceral regulatory patterns, thereby affecting intestinal blood flow, motility, secretion, mucosal immunity, and barrier homeostasis. Cohort studies of first-episode, drug-naïve MDD have shown that patients exhibit not only alterations in gut microbial composition, but also abnormal local functional activity in prefrontal regions, with certain microbial features associated with prefrontal functional indices and depression/anxiety scores (75). These findings suggest that depression-related brain network abnormalities may participate in the remodeling of the intestinal microenvironment through autonomic output. However, current evidence more strongly supports this as an associative framework of central regulation–intestinal response, rather than as a fully established single mechanistic pathway.

### 5.3 Descending remodeling of the gut microbial ecosystem by behavioral factors and medication exposure

In depressive disorders, changes in dietary patterns, circadian rhythm disruption, reduced physical activity, and increased exposure to smoking and alcohol accompanying the depressive state can also continuously reshape luminal substrate availability, intestinal transit, mucosal immunity, and the metabolic milieu, thereby amplifying microbial dysbiosis and its metabolic consequences (76). Therefore, within the MGBA framework, these factors should not be regarded merely as confounders, but rather as important behavioral and environmental effector layers through which brain states influence the intestinal microenvironment. Studies in adults have shown that social jetlag is associated with poorer diet quality and alterations in gut microbial composition, suggesting that circadian misalignment may affect the microbial ecosystem through both behavioral and metabolic pathways. Sedentary behavior and moderate-to-vigorous physical activity show opposite associations with a similar set of microbial taxa and functional pathways, including SCFA-related functional potential, indicating that reduced activity may serve as an important amplifying factor in the shift from depressive states toward an unfavorable microbial profile (77, 78).

**Figure 3:**
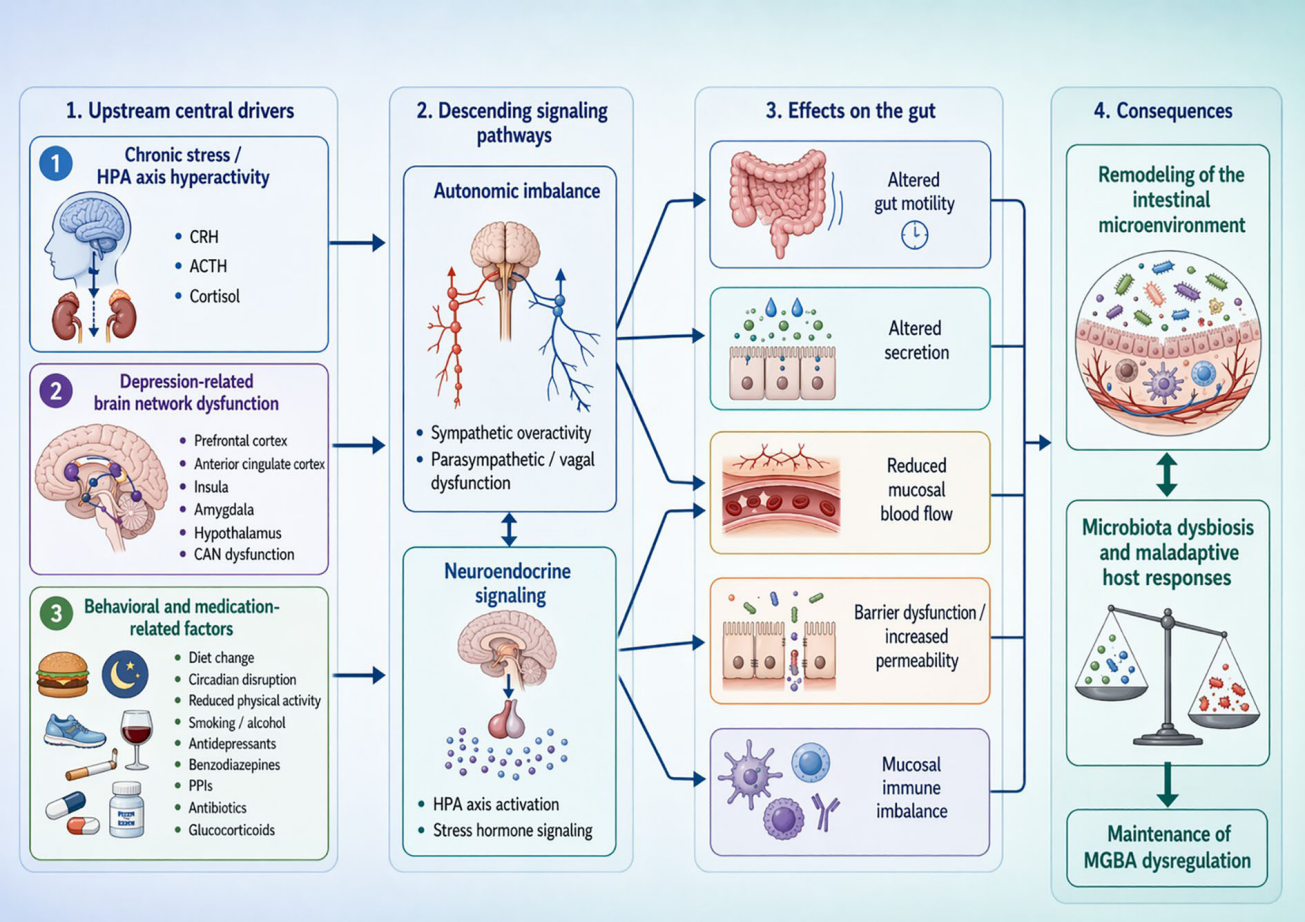
Integrated framework of brain-to-gut descending pathways in depressive disorders. This figure summarizes how chronic stress/HPA axis hyperresponsiveness, depression-related brain network abnormalities, and behavioral and medication exposure factors act on the gut through autonomic imbalance and neuroendocrine signaling, thereby influencing intestinal motility, secretion, mucosal blood flow, barrier function, and mucosal immune homeostasis, and promoting intestinal microenvironment remodeling, microbial dysbiosis, and the maintenance of maladaptive host responses. Arrows indicate the principal direction of action, whereas bidirectional arrows indicate interactions between processes.

Medication exposure is another important modulatory factor that may amplify the effects of brain-to-gut descending pathways, and it is of particular relevance in populations with depressive disorders. Compared with the general population, patients with depressive disorders are more likely to experience long-term, combined, or sequential medication use. Studies have shown that multiple classes of medications, including antidepressants, anxiolytics/benzodiazepines, proton pump inhibitors (PPIs), glucocorticoids, and antibiotics, are significantly associated with alterations in gut microbial composition and diversity (79, 80). Studies have shown that the microbiota-related effects of certain medications can remain detectable for several years after discontinuation, with evident carryover effects and additive effects. These medications include antidepressants, benzodiazepines or other psycholeptics, and glucocorticoids, among others (81). This suggests that in MGBA research, recording only medication use at the time of sampling may substantially underestimate the true impact of medications on microbial community structure. This issue may be particularly prominent in populations with psychiatric disorders. Studies have shown that antidepressant use is significantly associated with differences in gut microbial β-diversity, and its effect size may exceed that of certain diagnostic factors in overall analyses. Further stratified analyses indicate that some apparent disease-associated microbial differences are markedly attenuated after controlling for medication exposure (82). This suggests that many previously reported MDD-associated microbial features may have been confounded by medication-related signals. Thus, medication–microbiota interactions are not marginal phenomena, but important components influencing the interpretation of microbial ecological phenotypes, treatment responses, and therapeutic heterogeneity in patients with depressive disorders. Overall, the brain-to-gut descending pathway is not driven by any single factor, but rather results from the combined effects of chronic stress, brain network abnormalities, behavioral exposures, and medication-related factors. These upstream central drivers continuously act on intestinal motility, secretion, mucosal blood flow, barrier function, and local immune homeostasis through autonomic and neuroendocrine signaling, ultimately promoting intestinal microenvironment remodeling, microbial dysbiosis, and the maintenance of maladaptive host responses. **Figure 3** summarizes this brain-to-gut descending regulatory framework in depressive disorders.

## 6 Key metabolic hubs and integrative targets

### 6.1 Tryptophan metabolic network: TRP–KYN imbalance, KYNA/QUIN shift, and the indole branch

The tryptophan metabolic network occupies a central position in MGBA research on depressive disorders because it is not limited to the simple dichotomy of reduced 5-HT synthesis and enhanced KYN pathway activity. Rather, it encompasses the 5-HT branch, the kynurenine branch, and the indole branch directly involving gut microbial metabolism (23). The more informative question is no longer simply whether tryptophan metabolism shifts from 5-HT synthesis toward the KYN pathway, but rather how tryptophan is redistributed among different metabolic branches and whether an imbalance occurs between neurotoxic and neuroprotective downstream kynurenine metabolites.

In the context of chronic low-grade inflammation and HPA axis hyperresponsiveness, increased IDO and TDO activity promotes the diversion of tryptophan toward kynurenine metabolism, as reflected by reduced peripheral tryptophan availability and an elevated KYN/TRP ratio. This shift may translate immune activation and stress burden into depression-related neurobiological abnormalities (83). However, what may be more functionally informative for depressive phenotypes is often not the overall enhancement of the KYN pathway itself, but whether its downstream metabolic profile shifts toward the neurotoxic branch. Further metabolism of kynurenine can generate metabolites with divergent biological effects: some, such as KYNA, have relatively neuroprotective properties, whereas QUIN and 3-HK are more likely to participate in NMDA receptor-related excitotoxicity, oxidative stress, and glial activation (84, 85). Therefore, in depressive disorders, the key issue is not the absolute increase or decrease of any single metabolite, but whether the QUIN/KYNA balance shifts toward neurotoxicity and how this shift relates to symptom severity, cognitive impairment, and differences in treatment response. The role of the gut microbiota in this network lies not simply in the depletion or enrichment of a single substrate, but rather in reshaping the overall metabolic fate of tryptophan. On the one hand, microbial dysbiosis and intestinal barrier disruption can enhance local and systemic inflammation, promote IDO activation, and thereby drive tryptophan metabolism toward the kynurenine branch. On the other hand, gut microbes can directly participate in tryptophan metabolism by producing various indole derivatives, such as IPA, IAA, and ILA, thereby forming a microbiota-related branch that is not entirely dependent on host IDO/TDO activity (83, 86). Compared with the traditional TRP–KYN framework, the significance of the indole branch lies not merely in adding an additional category of metabolites, but in providing another mechanistic clue for understanding host–microbiota differences across depressive phenotypes. In some patients, phenotypic differences may not be explained solely by reduced 5-HT synthesis or enhanced KYN pathway activity, but may also be related to shifts in the overall allocation of tryptophan among the neurotoxic KYN branch, the protective KYN branch, and the microbial indole branch (87).

From an integrative MGBA perspective, the value of the tryptophan metabolic network lies in its ability to place inflammation, stress, barrier function, and neuroplasticity within a unified framework. Future stratified analyses integrating microbiome profiling, metabolomics, and clinical phenotypes may enable the tryptophan metabolic network to provide important clues for more mechanistically targeted monitoring and interventions in depressive disorders.

### 6.2 Short-chain fatty acids

SCFAs are core metabolites produced by the gut microbiota through the fermentation of dietary fiber, mainly including acetate, propionate, and butyrate. Unlike the tryptophan–kynurenine pathway, which more prominently reflects inflammatory activation and neurotoxic metabolic diversion, the key significance of SCFAs lies in their simultaneous effects on intestinal barrier homeostasis, mucosal immune regulation, host energy metabolism, and neuroplasticity. Therefore, SCFAs may be regarded as a homeostasis-maintaining hub that connects peripheral stability with central plasticity (88). Recent studies generally suggest that the protective effects of SCFAs are not confined to a single process, but extend across multiple levels of the MGBA. They can maintain epithelial barrier integrity, regulate immune tone, and influence brain function through neuroinflammatory and neurotrophic signaling (89). At the central level, one of the important mechanisms of SCFAs involves the regulation of neuroinflammation and neuroplasticity. Existing studies have shown that SCFAs may contribute to the maintenance of plasticity in brain regions such as the hippocampus and prefrontal cortex by inhibiting histone deacetylases (HDACs), modulating microglial activation, and influencing BDNF- and neurogenesis-related signaling (90). Recent human neurogenesis models further suggest that SCFAs can counteract IL-1β- and IL-6-induced reductions in neurogenesis and increases in apoptosis, indicating a direct protective role at the inflammation–neuroplasticity interface (91). In other words, the value of SCFAs lies not merely in their association with depressive disorders, but in their ability to integrate peripheral inflammatory buffering and central neuroplasticity protection within a unified mechanistic framework.

From the perspective of human evidence, SCFAs are among the few candidate metabolic markers that possess both mechanistic plausibility and practical measurability. A 2025 prospective study showed that circulating SCFA levels were associated with depression severity, and that baseline SCFA levels could predict MDD remission over a 6-month period (92). This suggests that SCFAs may not only participate in pathological processes, but also possess potential prognostic value. However, the direction of associations between specific SCFA subtypes and depressive phenotypes is not fully consistent across studies, indicating that their clinical interpretation should take into account background factors such as dietary patterns, BMI, metabolic status, and medication exposure (93, 94). Such inconsistency does not necessarily weaken the research value of SCFAs; rather, it suggests that SCFA-related findings are particularly susceptible to the combined influence of study design and host background. Differences across studies may arise from multiple factors, including sample type (feces, serum, or plasma), analytical platform, targeted SCFA subtypes, dietary status at sampling, obesity/metabolic comorbidity burden, and medication exposure. Therefore, a more appropriate future strategy is not to continue comparing a single SCFA as an isolated marker across studies, but to prioritize stratified study designs and conduct integrated analyses of fecal and circulating SCFA levels, SCFA-producing microbial features, and intestinal barrier/inflammatory indicators, with standardized recording of dietary and metabolic backgrounds. Only by placing SCFAs back into an integrated microbial function–host response framework can their heterogeneous patterns across different depressive subtypes be more accurately interpreted. Overall, SCFAs are better regarded as functional readouts within a combined intestinal barrier integrity–immune tone–neuroplasticity framework, rather than being simplified as single, universal biomarkers with fixed directional changes.

### 6.3 Bile acids, TMAO, vitamins, and related metabolites

Bile acids not only participate in lipid absorption, but also act as important signaling molecules. Within their receptor axes, the nuclear receptor FXR and the membrane receptor TGR5 are regarded as key hubs linking metabolism, inflammation, and neural function (95). Recent pathophysiological and mechanistic reviews have systematically summarized the roles of bile acid–FXR/TGR5 signaling in immune regulation, energy metabolism, and the nervous system, providing a clear theoretical basis for incorporating this pathway into the framework of psychiatric disorders. At the population level, existing studies suggest that patients with MDD, particularly those with a cognitive impairment phenotype, exhibit coupled features of gut microbial dysbiosis and abnormal bile acid metabolism. These findings further indicate that targeting the microbiota–bile acid metabolism axis may have potential therapeutic significance (25). At the animal level, recent studies have further linked bile acid signaling to stress susceptibility. Chronic social defeat stress (CSDS) may be accompanied by bile acid-related metabolic disturbances, while abnormal hippocampal TGR5 signaling is thought to contribute to depression-like behaviors and alterations in synaptic function (96). Thus, bile acids may not only reflect the background of metabolic comorbidity, but also participate in the regulation of inflammatory tone and central nervous system function through multiple pathways.

Trimethylamine N-oxide (TMAO) is a representative product of the dietary substrate–microbial metabolism–host oxidation axis and has long been primarily investigated in the context of cardiovascular and metabolic diseases. In relation to depressive disorders, TMAO more often appears as a metabolic signal associated with comorbidity risk and inflammatory amplification. It is closely coupled with factors such as metabolic syndrome, vascular risk, and sleep/inflammatory status, which themselves represent important sources of heterogeneity in depressive disorders (97). Recent studies in populations with post-stroke depression (PSD) and in animal models suggest that TMAO levels are associated with the severity of depressive symptoms. Further animal experiments have shown that TMAO can exacerbate pro-inflammatory responses and worsen depression-like behaviors, thereby providing more specific mechanistic clues for the TMAO–inflammation–affective phenotype axis (98). Vitamins, particularly folate and B vitamins, jointly regulate methyl donor availability, homocysteine levels, and the availability of neurotransmitter substrates through one-carbon metabolism, and have increasingly been incorporated into the diet–microbiota–inflammation–mood framework in recent years. Recent studies have indicated that certain vitamins are derived not only from the diet, but may also be synthesized or transformed by gut microbes. Alterations in these vitamins may indirectly influence mood disorders through immune regulation and neurotransmitter synthesis (99).

### 6.4 Hub-integrated model and monitorable targets

MGBA dysregulation associated with depressive disorders can be conceptualized as abnormalities in multiple interconnected functional nodes, including impaired intestinal barrier integrity and increased endotoxin translocation, elevated peripheral inflammatory tone, dysregulated stress-related neuroendocrine states, particularly abnormal HPA axis activation, disrupted outputs from key microbial metabolic hubs, and impaired neuroplasticity. This perspective helps consolidate dispersed mechanistic pathways into a limited set of trackable functional readouts and provides mechanistic endpoints for future interventional studies.

In translational research, a single biomarker is often insufficient to reliably characterize the state of this network. A more appropriate approach is to establish a combinatorial monitoring framework centered on intestinal barrier function, inflammatory tone, HPA axis status, and outputs from key metabolic hubs, and to interpret these readouts in the context of clinical backgrounds such as gastrointestinal symptom profiles, metabolic comorbidities, sleep/stress burden, and long-term medication use. **Table 2** summarizes candidate combinations of monitoring indicators for key functional nodes of the MGBA.

**Table 2:** Candidate combinations of monitoring indicators for key nodes of MGBA.

Functional node	Priority indicators	Optional supplementary indicators	References
Intestinal barrier integrity	I-FABP;zonulin	DAO; tight junction proteins	(40)
Endotoxin translocation/Innate immune activation	LBP or sCD14	LPS; anti-LPS IgA/IgM	(42)
Peripheral inflammatory tone	CRP;IL-6	TNF-α;IL-1β	(100)
HPA axis status	Morning cortisol peak or diurnal rhythm indices	ACTH; hair cortisol	(71)
Outputs from key metabolic hubs	KYN/TRP ratio; butyrate (± propionate); bile acid profile features, including the primary/secondary bile acid ratio or key secondary bile acids	QUIN/KYNA ratio; FXR/TGR5-related readouts	(18)

## 7 Interventions targeting the MGBA and clinical evidence

### 7.1 Dietary and lifestyle interventions

The advantages of dietary and lifestyle interventions within the MGBA framework lie in their upstream positioning and multi-target nature. By modifying substrate availability and the intestinal luminal ecological niche, these interventions can simultaneously influence microbial functional outputs, such as SCFAs, barrier homeostasis, and inflammatory tone, while also modulating stress reactivity and circadian rhythms to some extent, thereby affecting multiple depressive phenotypes, including mood, cognition, sleep, and somatic/gastrointestinal symptoms. Randomized controlled trials have shown that improving diet quality can significantly enhance symptom improvement in patients with moderate-to-severe depression, with higher remission rates observed in some studies. Therefore, dietary improvement may be regarded as a promising lifestyle intervention strategy for the adjunctive treatment of moderate-to-severe depression (101). Systematic reviews and meta-analyses further support that healthy dietary patterns, such as the Mediterranean diet, can improve depressive outcomes, and that their effect sizes may be influenced by intervention adherence, baseline metabolic risk, and inflammatory burden (102).

Evidence for exercise interventions is also moving toward the incorporation of mechanistic and microbiota-related endpoints. Randomized controlled trials have observed that aerobic exercise can improve depressive symptoms while being accompanied by changes in gut microbial composition, suggesting that exercise may act as a highly adaptable ecological niche modulator, with its effects partly mediated through microbial and metabolic pathways (103). Recent studies have further indicated that physical activity levels are associated with mild depressive states and microbial features, supporting the existence of detectable associations along the activity–microbiota–mood/brain function axis in real-world populations (104). It should be noted that different exercise prescriptions, including type, intensity, and duration, do not produce consistent directions of microbial change, which may partly explain the heterogeneity of the antidepressant effects of exercise. Therefore, higher-quality studies should simultaneously incorporate microbial and metabolic endpoints to enhance mechanistic interpretability (105).

Sleep and stress management are more closely aligned with the brain-to-gut descending pathway, but they may also influence the microbiota through circadian and inflammatory mediators. Animal and experimental studies have shown that sleep deprivation can disrupt circadian rhythmicity of the gut microbiota and is accompanied by disturbances in the rhythmic patterns of peripheral inflammatory cytokines, suggesting that circadian misalignment may amplify mood- and anxiety-related phenotypes through a microbial rhythmicity–inflammatory tone pathway (106). Large-scale cohort studies have also found that social jetlag is associated with gut microbial composition and dietary/metabolic features, underscoring the importance of regular sleep patterns and circadian alignment for the stability of the intestinal ecological niche (77).

Overall, dietary and exercise interventions act more directly on luminal substrates and microbial functional outputs, whereas sleep and stress management more directly target stress and circadian homeostasis. Combining these two types of strategies is therefore more consistent with the biological logic of bidirectional coupling within the MGBA. Current evidence also suggests that the effect sizes of lifestyle interventions are highly dependent on baseline phenotypes and exposure backgrounds. Therefore, incorporating phenotype-based stratification and microbiota/metabolism-related mechanistic endpoints into clinical trials and translational studies is a key strategy for improving reproducibility and interpretability.

### 7.2 Microbiota-based interventions: psychobiotics

In clinical research, psychobiotics primarily refer to microbiota-based therapeutic strategies, represented by probiotics, prebiotics, and synbiotics, that target emotional and cognitive outcomes; postbiotics are also gradually being incorporated into this research framework. Recent randomized controlled trials and systematic reviews suggest that these interventions may produce small-to-moderate improvements in depressive symptoms overall, although between-study heterogeneity remains substantial. Some trials have observed reductions in depression scale scores accompanied by improvements in anxiety symptoms, whereas others have not demonstrated significant between-group differences (107, 108). These findings suggest that therapeutic efficacy is closely related to baseline population characteristics, strain/substrate combinations, and the selection of outcome measures.

In populations with depressive disorders, one important line of evidence comes from studies using probiotics as an adjunctive intervention to standard antidepressant treatment. In an 8-week randomized controlled trial, multi-strain probiotics used as an adjunct to antidepressant therapy showed a greater trend toward symptom improvement than placebo, while acceptability and tolerability were systematically evaluated. These findings suggest that this strategy is feasible within clinical care pathways, although larger studies are needed to confirm its robust and reproducible effects (109). It should be noted that meta-analyses suggest that the depression rating scales used across different trials can substantially influence pooled conclusions. Taking microbiota-based interventions such as probiotics and synbiotics as an example, scale-stratified analyses may show significant improvement in subgroups assessed using the Beck Depression Inventory (BDI), whereas subgroups assessed using the depression subscale of the Hospital Anxiety and Depression Scale (HADS-D) or the Hamilton Depression Rating Scale (HAM-D) do not necessarily yield consistently significant results. This may partly explain the inconsistency of conclusions across different systematic reviews (110). Therefore, the overall effects of psychobiotics in depressive disorders appear to be generally positive, but are highly dependent on study design and population characteristics, and are not yet sufficient to support a universal prescription. Compared with probiotics, prebiotics and synbiotics place greater emphasis on regulating microbial function through substrate provision, particularly by influencing fermentation-related pathways such as SCFA production; therefore, their clinical effects may be more strongly affected by dietary patterns, metabolic background, and baseline microbiota status. Existing studies suggest that prebiotics and synbiotics may provide some benefit for depressive and anxiety symptoms, although between-study heterogeneity remains substantial. This indicates that future trials should systematically record dietary patterns, body weight/metabolic status, and medication history, and incorporate these factors into stratified or adjusted analyses (111). Evidence on postbiotics remains relatively recent and fragmented, and postbiotics are currently better regarded as an exploratory direction rather than a conclusive therapeutic strategy. Some clinical trials have begun to evaluate the effects of postbiotics on mood- or psychiatry-related outcomes; however, the study populations are often not limited to MDD, including, for example, critically ill populations, and both outcome measures and mechanistic endpoints remain insufficiently standardized (112). Therefore, more randomized controlled trials specifically targeting depressive disorders are needed, with simultaneous collection of microbiota and metabolic pathway readouts. In addition, existing psychobiotic trials commonly suffer from small sample sizes, substantial differences in strain combinations, insufficient control of baseline medication use and dietary intake, short follow-up durations, and inconsistent mechanistic endpoints, which are important reasons why findings across studies remain difficult to reproduce and corroborate consistently (113, 114).

### 7.3 Fecal microbiota transplantation and other microbiota reconstruction strategies

FMT more closely resembles a global reconstruction of the intestinal ecosystem; therefore, in research on depressive disorders, it is regarded not only as a potential intervention, but also as a tool for exploring whether depression-related microbial abnormalities have transferable biological effects and may provide clues to causal relevance. Existing preclinical studies have shown that transplantation of microbiota from patients with depression into recipient animals can induce more pronounced depression-like behaviors, such as increased immobility time and reduced struggling behavior in the forced swim test. Some studies have further observed that, compared with animals receiving microbiota from healthy donors, those receiving depression-derived microbiota exhibit divergence in certain microbial members and related metabolic phenotypes. This suggests that these effects are not merely nonspecific stimuli induced by transplantation itself, but may reflect differences in the functional status of donor microbiota (5, 6). The significance of these findings does not lie simply in determining whether FMT can treat depression, but rather in advancing depression-related microbial abnormalities from a correlative phenomenon to the level of a transferable biological phenotype. Therefore, FMT-related findings are better interpreted as important clues that depression-related microbial abnormalities may exert transferable biological effects, rather than as direct evidence at the population level that they represent a primary etiological cause.

At the population level, evidence for the use of FMT in depressive disorders remains limited, but noteworthy early clinical signals have begun to emerge. Early case reports of patients with major depressive disorder receiving FMT as an adjunctive treatment showed reductions in depression scores as early as 4 weeks after transplantation. In two patients, Beck Depression Inventory-II (BDI-II) scores decreased from 50 to 31 and from 24 to 12, respectively; at the 8-week follow-up, these improvements were partially maintained, accompanied by alleviation of gastrointestinal symptoms (115). This suggests that FMT may not act solely on mood-related scores, but may also indirectly reduce the overall disease burden by improving gastrointestinal symptoms such as constipation and abdominal bloating. More recent randomized studies have begun to show short-term signals of enhanced efficacy. A randomized study involving 46 patients with depressive episodes showed that, after 2 weeks, the FMT combined with pharmacotherapy group had a greater reduction in 24-item Hamilton Depression Rating Scale (HAMD-24) scores and a higher score reduction rate than the pharmacotherapy-alone group. Meanwhile, after FMT, increased levels of *Enterococcus*, *Lactobacillus*, *Bifidobacterium*, and *Butyricicoccus* were observed. Correlation analysis further suggested that baseline enrichment of *Faecalibacterium prausnitzii* and *Eubacterium rectale* was associated with lower HAMD-24 scores (116). This suggests that the potential benefits of FMT may not be limited to microbial supplementation, but may more likely involve the reconstruction of SCFA-producing bacteria and commensal taxa associated with ecological homeostasis. Overall, research on FMT in depressive disorders remains at an early exploratory stage. Existing evidence suggests that FMT provides important clues for evaluating the transferability and causal relevance of depression-related microbial abnormalities, but remains insufficient to directly establish them as a primary etiological cause at the population level. At the same time, FMT may improve depressive symptoms and gastrointestinal discomfort in some patients by reconstructing SCFA-producing and homeostasis-associated commensal bacteria. However, current clinical studies still have small sample sizes and short follow-up durations, and the efficacy and safety of FMT require further confirmation in more high-quality studies. In terms of the current evidence base, population-level data on FMT are still mainly derived from case reports, small single-center studies, and short-term randomized trials. Moreover, donor screening, transplantation protocols, concomitant treatment backgrounds, and outcome assessment methods remain insufficiently standardized; therefore, FMT is currently better regarded as an exploratory strategy with mechanistic implications, rather than a routine intervention with a fully mature evidence base.

### 7.4 Heterogeneity in the efficacy of gut microbiota-targeted interventions

Existing studies suggest that interventions targeting MGBA have overall antidepressant potential, but their therapeutic effects are not uniform and show substantial heterogeneity. Meta-analyses have shown that microbiome-targeted therapies may improve depressive symptoms to some extent; however, the overall effect sizes are limited, and substantial heterogeneity exists across studies in terms of population characteristics, intervention types, strain combinations, treatment duration, and outcome assessment (117). These findings suggest that such interventions are more likely to be applicable to specific patient subgroups, rather than constituting a universal standardized regimen.

Based on current evidence, patients with depressive disorders who present with prominent gastrointestinal symptoms, metabolic abnormalities, or low-grade inflammatory states may be more likely to represent priority responder subgroups for MGBA-targeted interventions. These patients often exhibit concurrent intestinal barrier disruption, microbial metabolic abnormalities, and immune-inflammatory activation, suggesting that gut microbial dysbiosis may play a more prominent role in their disease process and that they may, in theory, be more responsive to microbiome-targeted interventions. However, this assumption is still based mainly on mechanistic inference, secondary outcomes, and subgroup signals, and requires validation in further prospective stratified studies (118). Meanwhile, existing studies suggest that patients who achieve remission and those who do not may already differ in their baseline microbial features. In older adults with depression, a prospective pilot study showed that a baseline microbiota-based random forest model constructed using nine genera achieved an AUC of 0.857 for predicting remission. In this model, baseline enrichment of SCFA-producing taxa such as *Faecalibacterium*, *Agathobacter*, and *Roseburia* was associated with subsequent remission (119). Therefore, it can be hypothesized that treatment efficacy may be influenced not by broadly defined microbial dysbiosis per se, but by whether patients possess a potentially modifiable microbiota-responsive phenotype.

This heterogeneity is also evident across different intervention modalities. Dietary and lifestyle interventions are more suitable as long-term management strategies and foundational background interventions; psychobiotics, including probiotics, prebiotics, and synbiotics, generally exert relatively modest symptom-improving and modulatory effects; whereas FMT and other microbiota reconstruction strategies may be more appropriate for populations with more pronounced microbial dysbiosis who require stronger ecological remodeling (120). A study involving 46 patients with depressive episodes showed that, after 2 weeks, the FMT combined with pharmacotherapy group had a greater reduction in HAMD-24 scores and a higher score reduction rate than the pharmacotherapy-alone group, accompanied by increased levels of *Lactobacillus*, *Bifidobacterium*, and *Butyricicoccus (*116). Although these findings suggest that more intensive microbiota reconstruction may provide additional benefits for some patients, the current evidence is still mainly derived from small-sample studies with short follow-up durations. Therefore, at the current stage, gut microbiota-targeted interventions are better positioned as stratified and individualized adjunctive therapeutic strategies, and their clinical application still requires careful balancing between potential benefits and safety boundaries.

## 8 Future challenges and directions

### 8.1 Further elucidation of causal mechanisms and key pathways

Although research on MGBA and depressive disorders has increased substantially in recent years, the current evidence remains largely dominated by correlative descriptions, and a stable and clearly defined causal chain has not yet been established. Existing studies suggest that patients with depressive disorders are often accompanied by gut microbial dysbiosis, abnormalities in key metabolites, inflammatory activation, and neuroendocrine dysregulation. Meanwhile, animal experiments and FMT studies have, to some extent, supported the transferability of depression-related microbial abnormalities and associated phenotypes (12). However, these findings more strongly indicate that the gut microbiota participates in the onset and progression of depressive disorders, rather than simply proving that it occupies an equally central etiological position in all patients. In other words, the gut microbiota is more likely to serve as an important modulatory factor within the pathological network of depressive disorders, rather than as a single independent cause.

Future research should further focus on the integrative interpretation of key pathways. Current evidence indicates that SCFAs, the tryptophan–kynurenine pathway, bile acid signaling, intestinal barrier disruption, and neuroinflammatory responses remain key directions worthy of continued investigation in research on the MGBA and depressive disorders (121). Compared with merely comparing differences in microbial composition, these functional and metabolic pathways are closer to key nodes linking microbial alterations, host responses, brain functional abnormalities, and depressive phenotypes, and therefore have greater value for mechanistic interpretation and clinical translation. In particular, SCFAs exert multiple effects in maintaining intestinal barrier homeostasis, regulating immune-inflammatory responses, and influencing brain plasticity, whereas shifts in tryptophan metabolism link microbial alterations to neurotransmitter imbalance and the accumulation of neurotoxic metabolites. These findings suggest that different pathways may not operate in isolation, but instead jointly participate in the onset and progression of depressive disorders. At the same time, the interrelationships among these pathways require further clarification. Many current studies explain the relationship between the MGBA and depressive disorders from separate perspectives, such as inflammation, the HPA axis, neurotransmitters, or the intestinal barrier. However, in real disease processes, these mechanisms are often intertwined and mutually amplifying, rather than existing in isolation. Future studies should use longitudinal designs and multi-level integrative analyses to further clarify the relative contributions of different key pathways at different stages of disease. This will help gradually establish a more continuous and reliable mechanistic evidence chain, providing a stronger theoretical basis for subsequent biomarker screening and precision interventions.

### 8.2 Multi-omics integration and the establishment of a standardized research framework

Another key issue in current research on the MGBA and depressive disorders is the lack of sufficiently unified standards across studies in terms of sample inclusion, technical approaches, and analytical frameworks, which is an important contributor to the limited reproducibility of findings. Taking microbiome research itself as an example, differences in multiple steps, ranging from fecal sample collection time, storage conditions, and DNA extraction methods to sequencing platforms, bioinformatics pipelines, and statistical strategies, may all influence the final results. Meanwhile, 16S rRNA sequencing, metagenomic sequencing, and 16S-based functional prediction analyses each have limitations in resolution and depth of information, meaning that different studies may obtain inconsistent microbial features even when investigating similar populations (122). Similar issues are also present in metabolomic studies. Differences in analytical platforms, such as targeted and untargeted metabolomics, LC–MS, and GC–MS, as well as sample pretreatment methods, quantification strategies, and metabolite annotation standards, may all lead to inconsistent metabolite profile features for the same study population across different studies (123, 124).

On the other hand, reliance solely on taxonomic information of the gut microbiota has become increasingly insufficient for explaining the complex pathological processes of depressive disorders. Compared with static descriptions of which taxa increase or decrease, abnormalities at the functional and metabolic levels are often more stable and are closer to the key links connecting microbial alterations, host responses, brain functional abnormalities, and depressive phenotypes. SCFAs, the tryptophan–kynurenine pathway, bile acid signaling, and inflammatory responses all suggest that the influence of the gut microbiota on depressive disorders is not reflected merely in changes in community composition, but is more likely mediated through multi-level mechanisms, including metabolic remodeling, immune regulation, and neuroendocrine dysregulation (121). Therefore, a more valuable future direction is to integrate the microbiome with metabolomics, inflammatory indicators, endocrine status, neuroimaging data, and genetic/epigenetic information, thereby establishing a multi-level integrative framework centered on key functional axes. At the same time, multi-omics integration does not merely involve increasing the dimensionality of data; more importantly, it requires an appropriate study design that can meaningfully link information across different biological levels. For depressive disorders, factors such as diet, medication use, sleep, metabolic status, and gastrointestinal comorbidities may simultaneously influence microbial, metabolic, and inflammatory outcomes. Without systematic recording and adjustment for these variables, even large-scale omics datasets may fail to yield stable and biologically interpretable signals. Overall, the establishment of multi-omics integration and standardized research frameworks is an essential prerequisite for advancing MGBA–depression research from descriptive associations toward mechanistic integration and clinical translation. Only by standardizing research procedures, improving technical comparability, strengthening functional readouts, and integrating clinical phenotypes can research findings be more effectively translated into stable and reproducible biomarkers and clinically meaningful intervention targets.

### 8.3 Clinical translation of stratified identification and precision intervention

**Figure 4:**
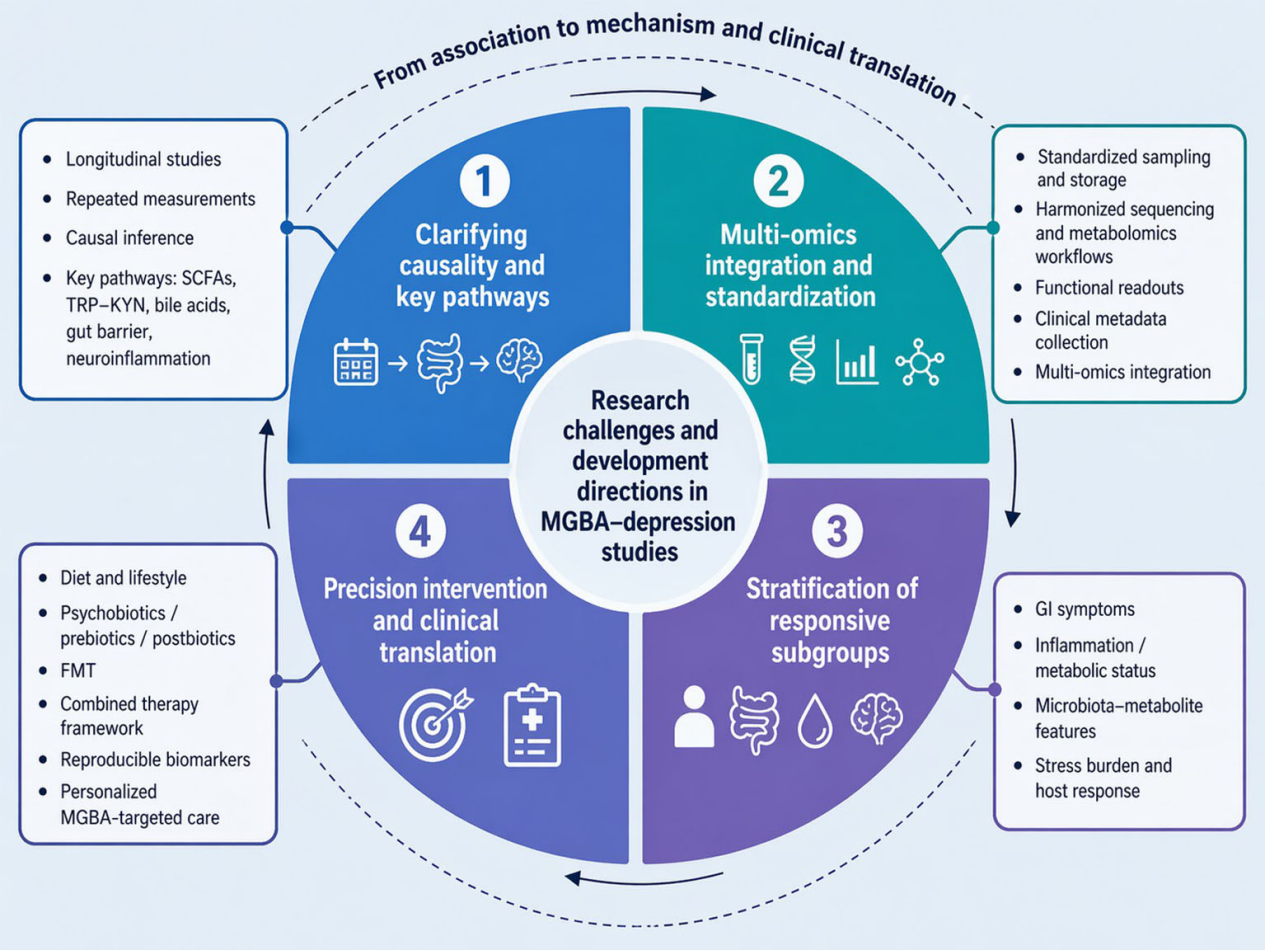
An integrative framework of the research challenges and future directions in MGBA–depression research. This figure summarizes the major future research priorities in this field from four perspectives: elucidation of causal mechanisms and key pathways, multi-omics integration and standardization, stratified identification of responder subgroups, and precision intervention with clinical translation. It further emphasizes that research should continue to advance along the trajectory from correlation to mechanism and clinical translation. Arrows indicate the main directions of research progression.

As research on the MGBA continues to deepen, the future focus of clinical translation is no longer simply to determine whether gut microbiota-targeted interventions are effective, but to identify which patients are more likely to benefit and to develop more targeted intervention strategies accordingly. Depressive disorders are inherently highly heterogeneous, with substantial interindividual differences in symptom spectra, gastrointestinal comorbidities, inflammatory levels, metabolic status, and microbial functional features (125). This also means that patients may differ in their responsiveness to interventions such as dietary modification, probiotics/prebiotics, postbiotics, and FMT. Therefore, a more rational future direction is to move beyond crude comparisons between patients with depressive disorders and healthy controls, and toward stratified identification based on clinical phenotypes and microbial ecological features. On this basis, the implementation of precision intervention depends on the integrated assessment of symptom profiles, comorbidity burden, and key biological indicators. For example, patients with prominent gastrointestinal symptoms, metabolic abnormalities, or low-grade inflammatory states are more likely to exhibit intestinal barrier disruption, microbial metabolic abnormalities, and immune-inflammatory activation, and may therefore deserve priority consideration in evaluations of MGBA-targeted interventions. However, this judgment is still based mainly on mechanistic inference, secondary outcomes, and subgroup signals, and requires validation in further prospective stratified studies. Meanwhile, existing studies suggest that patients who achieve remission and those who do not may already differ in their baseline microbial features. This suggests that treatment efficacy may be influenced not by broadly defined microbial dysbiosis per se, but by whether patients possess a potentially modifiable microbiota-related phenotype (126). It should be further emphasized that a “microbiota-responsive phenotype” should not be simply understood as an alternative term for a “high-stress phenotype.” For this issue, a more appropriate population-level identification framework should include at least three dimensions. First, temporal precedence should be assessed through longitudinal follow-up and repeated measurements to determine whether microbial and metabolic abnormalities precede symptom fluctuations or primarily change in parallel with stress burden and disease status. Second, interventional reversibility should be evaluated by determining whether interventions targeting the microbiota or metabolic pathways can induce biological reversal consistent with symptom improvement in specific subgroups. Third, stratification specificity should be examined by assessing whether subgroups defined by gastrointestinal symptoms, metabolic/inflammatory status, and microbiota–metabolite features retain independent predictive value after controlling for stress burden, HPA axis status, medication exposure, diet, and related factors. Only on this basis can a microbiota-responsive phenotype be more reliably distinguished from a simple high-stress phenotype. Overall, the future development of MGBA–depression research does not lie merely in continuing to accumulate evidence of microbial differences, but in advancing along the main trajectory of causal mechanism elucidation, multi-omics integration and standardization, stratified identification of priority populations, and precision intervention with clinical translation. Only by simultaneously improving mechanistic understanding, methodological rigor, and clinical stratification can research findings be more effectively translated into stable and reproducible biomarkers and intervention strategies with practical clinical value. **Figure 4** summarizes the overall framework of these research challenges and future directions.

## 9 Discussion

Taken together, existing preclinical and clinical studies indicate that MGBA provides a more integrative biological framework for understanding depressive disorders than traditional single-pathway models. Based on the current strength of evidence, the MGBA is better understood as an amplifier and regulator in depressive disorders, rather than as an established single upstream cause. Dysregulation of gut microbial composition and function, reduced intestinal barrier integrity, persistent peripheral inflammatory activation, HPA axis abnormalities, altered vagal signaling, and impairments in neurotransmitter systems and neuroplasticity are not isolated parallel phenomena; rather, they are more likely to constitute an interconnected and self-amplifying pathological network. This may explain why gastrointestinal symptoms, metabolic abnormalities, chronic low-grade inflammation, and cognitive–affective disturbances commonly coexist in depressive disorders and can be interpreted within a unified mechanistic framework. Rather than simply conceptualizing depressive disorders as a consequence of monoamine deficiency or abnormalities in a single brain region, they may be more appropriately viewed as the result of multi-system dysregulation across brain, gut, immune, and metabolic networks. The MGBA provides a key interface through which this dysregulation can be observed, monitored, and potentially targeted.

At the same time, however, the MGBA should not be overgeneralized as a single etiological cause of depressive disorders. A more reasonable interpretation is that the MGBA functions more as an important regulator and amplifier in the onset and progression of depressive disorders, rather than as the sole core mechanism capable of explaining all symptoms in all patients. Depressive disorders are inherently heterogeneous, with marked interindividual differences in gastrointestinal symptoms, metabolic comorbidities, inflammatory burden, sleep rhythms, and psychosocial stress exposure, which means that the pathological weight of microbiota-related mechanisms may vary across individuals. Some patients may be dominated by abnormalities in the gut-to-brain pathway, characterized more prominently by intestinal barrier disruption, increased low-grade inflammation, key metabolite imbalance, and accompanying gastrointestinal symptoms. Others may be dominated by abnormalities in brain-to-gut descending regulation, in which stress–HPA axis–autonomic imbalance further amplifies symptoms by altering intestinal motility, secretion, and the microbial ecological niche. From this perspective, the greatest value of the MGBA does not lie in assigning the same microbiota-modulating indication to all patients with depressive disorders, but in helping to identify different dominant pathological directions and distinct microbiota-related subtypes.

From the interface between mechanistic understanding and clinical translation, what holds real clinical promise is not a fragmented list of differential taxa, but a more operational stratification framework built around key functional axes and metabolic hubs. The tryptophan–kynurenine pathway, SCFAs, bile acid signaling, TMAO, and related metabolites deserve attention not only because they are associated with depressive symptoms, but also because they connect multiple levels, including intestinal barrier status, inflammatory tone, HPA axis activity, neurotoxic metabolism, and changes in neuroplasticity. In particular, SCFAs combine mechanistic plausibility with practical measurability, making them more suitable as functional readouts within an integrated intestinal barrier integrity–immune tone–neuroplasticity framework. Bile acids and TMAO, in contrast, highlight the important role of metabolic comorbidity backgrounds in the heterogeneity of depressive disorders. Compared with simply comparing which taxa increase or decrease, these functional and metabolic indicators are closer to candidate biomarkers for future risk stratification, treatment response prediction, and relapse warning. They may also provide more practical entry points through which dietary interventions, psychobiotics, FMT, and other microbiota reconstruction strategies can realize their value in precision intervention.

From the perspective of clinical practice, MGBA-targeted interventions should not be regarded as a new “universal solution” that replaces existing antidepressant treatments, but rather as an adjunctive pathway that complements pharmacotherapy, psychotherapy, and lifestyle management. Patients with prominent gastrointestinal symptoms, metabolic abnormalities, or low-grade inflammatory states may be more likely to exhibit microbiota-related features and to benefit from gut microbiota-targeted interventions. In contrast, for patients dominated by marked psychosocial stress, sleep–circadian rhythm disturbances, or cognitive symptoms, microbiota-based interventions should be considered within a broader integrated treatment framework. This suggests that the future priority should not be the mechanical repetition of the same microbiota-modulating treatment for all patients with depressive disorders, but rather individualized selection after stratification based on symptom profiles, comorbidity burden, and key biological indicators. Only when the question of “who is more likely to benefit” is answered more clearly can MGBA-related interventions move from a research hotspot toward clinically meaningful precision strategies.

Nevertheless, this field remains some distance from routine clinical implementation. On the one hand, existing studies still vary substantially in diagnostic criteria, enrolled populations, medication exposure, dietary patterns, sleep status, and control of comorbidities, while the high proportion of small-sample and cross-sectional studies limits causal inference and reproducibility. On the other hand, inconsistencies in sample collection, storage conditions, sequencing platforms, and analytical pipelines in microbiome research also make direct comparisons across studies difficult. Although some interventional studies have shown positive signals, the overall evidence is still characterized by limited average effect sizes and unclear target populations, and remains insufficient to support excessive extrapolation in the form of a “microbiota panacea” narrative. Therefore, evaluation of the MGBA should recognize its potential value in mechanistic integration and stratified treatment, while maintaining methodological restraint and avoiding the premature translation of correlative findings into definitive clinical conclusions.

In summary, research on MGBA is driving a shift in the conceptualization of depressive disorders from a traditional brain-centered model toward an integrated brain–gut–immune–metabolic model. Its true significance does not lie in proposing a new single hypothesis to replace existing theories, but rather in providing a more systematic explanatory framework for understanding the clinical heterogeneity, psychosomatic comorbidity features, and individualized interventions of depressive disorders. In the future, only on the basis of more rigorous methodological designs, more stable functional and metabolic readouts, and clearer patient stratification can the MGBA move beyond a mechanistic research hotspot and become a usable tool and actionable target within precision diagnosis and treatment pathways for depressive disorders.

## References

[1] Mehta I Juneja K Nimmakayala T Bansal L Pulekar S Duggineni D . Gut microbiota and mental health: A comprehensive review of gut-brain interactions in mood disorders. Cureus. (2025) 17:e81447. doi: 10.7759/cureus.81447 40303511 PMC12038870

[2] Sanada K Nakajima S Kurokawa S Barceló-Soler A Ikuse D Hirata A . Gut microbiota and major depressive disorder: A systematic review and meta-analysis. J Affect Disord. (2020) 266:1–13. doi: 10.1016/j.jad.2020.01.102 32056863

[3] Loh JS Mak WQ Tan LKS Ng CX Chan HH Yeow SH . Microbiota–gut–brain axis and its therapeutic applications in neurodegenerative diseases. Signal Transduction Targeted Ther. (2024) 9:37. doi: 10.1038/s41392-024-01743-1 38360862 PMC10869798

[4] Liu L Wang H Chen X Zhang Y Zhang H Xie P . Gut microbiota and its metabolites in depression: From pathogenesis to treatment. EBioMedicine. (2023) 90:104527. doi: 10.1016/j.ebiom.2023.104527 36963238 PMC10051028

[5] Knudsen JK Michaelsen TY Bundgaard-Nielsen C Nielsen RE Hjerrild S Leutscher P . Faecal microbiota transplantation from patients with depression or healthy individuals into rats modulates mood-related behaviour. Sci Rep. (2021) 11:21869. doi: 10.1038/s41598-021-01248-9 34750433 PMC8575883

[6] Kelly JR Borre Y O'Brien C Patterson E El Aidy S Deane J . Transferring the blues: Depression-associated gut microbiota induces neurobehavioural changes in the rat. J Psychiatr Res. (2016) 82:109–18. doi: 10.1016/j.jpsychires.2016.07.019 27491067

[7] Zhang Y Lai W Wang M Lai S Liu Q Luo Q . Gut microbial metabolic disorder in depression: Insights from computational modeling and mediation analysis. BMC Microbiol. (2026). doi: 10.1101/2024.10.10.617513 41975251 PMC13203000

[8] Zhang YW Zhao SS Li XY Liu HZ . Blood metabolites mediate gut microbiota effects on depression: A Mendelian randomization study. J Affect Disord. (2026) 394:120478. doi: 10.1016/j.jad.2025.120478 41197911

[9] Zhou M Fan Y Xu L Yu Z Wang S Xu H . Microbiome and tryptophan metabolomics analysis in adolescent depression: Roles of the gut microbiota in the regulation of tryptophan-derived neurotransmitters and behaviors in human and mice. Microbiome. (2023) 11:145. doi: 10.1186/s40168-023-01589-9 37386523 PMC10311725

[10] Cao Y Cheng Y Pan W Diao J Sun L Meng M . Gut microbiota variations in depression and anxiety: A systematic review. BMC Psychiatry. (2025) 25:443. doi: 10.1186/s12888-025-06871-8 40312666 PMC12044767

[11] Li X Jing K Lu H Li K Zhang Y Hasichaolu . Exploring the correlation between changes in gut microbial community diversity and depression in human populations. BioMed Res Int. (2022) 2022:6334868. doi: 10.1155/2022/6334868 35937392 PMC9355758

[12] Gao M Wang J Liu P Tu H Zhang R Zhang Y . Gut microbiota composition in depressive disorder: A systematic review, meta-analysis, and meta-regression. Transl Psychiatry. (2023) 13:379. doi: 10.1038/s41398-023-02670-5 38065935 PMC10709466

[13] Jiang H Ling Z Zhang Y Mao H Ma Z Yin Y . Altered fecal microbiota composition in patients with major depressive disorder. Brain Behavior Immun. (2015) 48:186–94. doi: 10.1016/j.bbi.2015.03.016 25882912

[14] Valles-Colomer M Falony G Darzi Y Tigchelaar EF Wang J Tito RY . The neuroactive potential of the human gut microbiota in quality of life and depression. Nat Microbiol. (2019) 4:623–32. doi: 10.1038/s41564-018-0337-x 30718848

[15] Nikolova VL Smith MRB Hall LJ Cleare AJ Stone JM Young AH . Perturbations in gut microbiota composition in psychiatric disorders: A review and meta-analysis. JAMA Psychiatry. (2021) 78:1343–54. doi: 10.1001/jamapsychiatry.2021.2573 34524405 PMC8444066

[16] Lai WT Deng WF Xu SX Zhao J Xu D Liu YH . Shotgun metagenomics reveals both taxonomic and tryptophan pathway differences of gut microbiota in major depressive disorder patients. Psychol Med. (2021) 51:90–101. doi: 10.1017/s0033291719003027 31685046

[17] Xie Z Huang J Sun G He S Luo Z Zhang L . Integrated multi-omics analysis reveals gut microbiota dysbiosis and systemic disturbance in major depressive disorder. Psychiatry Res. (2024) 334:115804. doi: 10.1016/j.psychres.2024.115804 38417224

[18] Jia M Fan Y Ma Q Yang D Wang Y He X . Gut microbiota dysbiosis promotes cognitive impairment via bile acid metabolism in major depressive disorder. Transl Psychiatry. (2024) 14:503. doi: 10.1038/s41398-024-03211-4 39719433 PMC11668851

[19] Chen H Li C Peng X Zhou Z Weinstein JN Cancer Genome Atlas Research Network . A pan-cancer analysis of enhancer expression in nearly 9000 patient samples. Cell. (2018) 173:386–399.e312. doi: 10.1016/j.cell.2018.03.027 29625054 PMC5890960

[20] Yu S Wang L Jing X Wang Y An C . Features of gut microbiota and short-chain fatty acids in patients with first-episode depression and their relationship with the clinical symptoms. Front Psychol. (2023) 14:1088268. doi: 10.3389/fpsyg.2023.1088268 37168424 PMC10165121

[21] Cheng J Hu H Ju Y Liu J Wang M Liu B . Gut microbiota-derived short-chain fatty acids and depression: Deep insight into biological mechanisms and potential applications. Gen Psychiatry. (2024) 37:e101374. doi: 10.1136/gpsych-2023-101374 38390241 PMC10882305

[22] Zhu X Hu J Deng S Tan Y Qiu C Zhang M . Comprehensive bibliometric analysis of the kynurenine pathway in mood disorders: Focus on gut microbiota research. Front Pharmacol. (2021) 12:687757. doi: 10.3389/fphar.2021.687757 34239441 PMC8258344

[23] Lukić I Ivković S Mitić M Adžić M . Tryptophan metabolites in depression: Modulation by gut microbiota. (2022) 16:987697. 10.3389/fnbeh.2022.987697 PMC9510596 36172468

[24] Jiang H Chen C Gao J . Extensive summary of the important roles of indole propionic acid, a gut microbial metabolite in host health and disease. (2023) 15:151. doi: 10.3390/nu15010151 PMC9824871 36615808

[25] Sun N Zhang J Wang J Liu Z Wang X Kang P . Abnormal gut microbiota and bile acids in patients with first-episode major depressive disorder and correlation analysis. Psychiatry Clin Neurosci. (2022) 76:321–8. doi: 10.1111/pcn.13368 35445772

[26] Clerici L Bottari D Bottari B . Gut microbiome, diet and depression: Literature review of microbiological, nutritional and neuroscientific aspects. Curr Nutr Rep. (2025) 14:30. doi: 10.1007/s13668-025-00619-2 39928205 PMC11811453

[27] Khawagi WY Al-Kuraishy HM Hussein NR Al-Gareeb AI Atef E Elhussieny O . Depression and type 2 diabetes: A causal relationship and mechanistic pathway. Diabetes Obes Metab. (2024) 26:3031–44. doi: 10.1111/dom.15630 38802993

[28] Cao B Lu C Yan L McIntyre RS Teopiz KM Lu Z . The dysbiosis of gut microbiota in major depressive disorder and comorbidity with overweight/obesity: Unraveling biomarkers and metabolic pathways from a microbial perspective. BMC Psychiatry. (2025) 25:1069. doi: 10.1186/s12888-025-07388-w 41204410 PMC12595788

[29] Tan R Han C Sun D Zhang X Liu T Sun H . Research progress on the mechanisms of comorbidity between functional gastrointestinal disorders and mental disorders: A review. Med (Baltimore). (2025) 104:e42925. doi: 10.1097/md.0000000000042925 40696692 PMC12282835

[30] Mujagic Z Kasapi M Jonkers DM Garcia-Perez I Vork L Weerts ZZRM . Integrated fecal microbiome-metabolome signatures reflect stress and serotonin metabolism in irritable bowel syndrome. Gut Microbes. (2022) 14:2063016. doi: 10.1080/19490976.2022.2063016 35446234 PMC9037519

[31] Carabotti M Scirocco A Maselli MA Severi C . The gut-brain axis: Interactions between enteric microbiota, central and enteric nervous systems. Ann Gastroenterol. (2015) 28:203–9. PMC4367209 25830558

[32] Kaelberer MM Buchanan KL Klein ME Barth BB Montoya MM Shen X . A gut-brain neural circuit for nutrient sensory transduction. Science. (2018) 361:eaat5236. doi: 10.1126/science.aat5236 30237325 PMC6417812

[33] Bonaz B Bazin T Pellissier S . The vagus nerve at the interface of the microbiota-gut-brain axis. (018) 12:49. doi: 10.3389/fnins.2018.00049 PMC5808284 29467611

[34] Margolis KG Cryan JF Mayer EA . The microbiota-gut-brain axis: From motility to mood. Gastroenterology. (2021) 160:1486–504. doi: 10.1053/j.gastro.2020.10.066 33493503 PMC8634751

[35] Holmberg SM Feeney RH Prasoodanan PV Puértolas-Balint F Singh DK Wongkuna S . The gut commensal Blautia maintains colonic mucus function under low-fiber consumption through secretion of short-chain fatty acids. Nat Commun. (2024) 15:3502. doi: 10.1038/s41467-024-47594-w 38664378 PMC11045866

[36] Pontarollo G Kollar B Mann A Khuu MP Kiouptsi K Bayer F . Commensal bacteria weaken the intestinal barrier by suppressing epithelial neuropilin-1 and Hedgehog signaling. Nat Metab. (2023) 5:1174–87. doi: 10.1038/s42255-023-00828-5 37414930 PMC10365997

[37] Kano M Muratsubaki T Van Oudenhove L Morishita J Yoshizawa M Kohno K . Altered brain and gut responses to corticotropin-releasing hormone (CRH) in patients with irritable bowel syndrome. Sci Rep. (2017) 7:12425. doi: 10.1038/s41598-017-09635-x 28963545 PMC5622133

[38] Neurath MF Artis D Becker C . The intestinal barrier: A pivotal role in health, inflammation, and cancer. Lancet Gastroenterol Hepatol. (2025) 10:573–92. doi: 10.1016/s2468-1253(24)00390-x 40086468

[39] Zhao J Bi W Xiao S Lan X Cheng X Zhang J . Neuroinflammation induced by lipopolysaccharide causes cognitive impairment in mice. Sci Rep. (2019) 9:5790. doi: 10.1038/s41598-019-42286-8 30962497 PMC6453933

[40] Morena D Lippi M Scopetti M Turillazzi E Fineschi V . Leaky gut biomarkers as predictors of depression and suicidal risk: A systematic review and meta-analysis. (2025) 15:1683. doi: 10.3390/diagnostics15131683 PMC12249198 40647682

[41] Ataei P Kalantari H Bodnar TS Turner RJ . The gut–brain connection: Microbes’ influence on mental health and psychological disorders. (2026) 4:1701608. doi: 10.3389/frmbi.2025.1701608 PMC12993674 41852385

[42] Zhu Z Cheng Y Liu X Xu X Ding W Ling Z . The microbiota-gut-brain axis in depression: Unraveling the relationships and therapeutic opportunities. (2025) 16:1644160. doi: 10.3389/fimmu.2025.1644160 PMC12507892 41080562

[43] Evrensel A Ünsalver B Ceylan ME . Neuroinflammation, gut-brain axis and depression. Psychiatry Investig. (2020) 17:2–8. doi: 10.30773/pi.2019.08.09 31587531 PMC6992852

[44] Hassamal S . Chronic stress, neuroinflammation, and depression: An overview of pathophysiological mechanisms and emerging anti-inflammatories. (2023) 14:1130989. doi: 10.3389/fpsyt.2023.1130989 PMC10213648 37252156

[45] Klose CSN Artis D . Innate lymphoid cells control signaling circuits to regulate tissue-specific immunity. Cell Res. (2020) 30:475–91. doi: 10.1038/s41422-020-0323-8 32376911 PMC7264134

[46] Zhang W Rutlin J Eisenstein SA Wang Y Barch DM Hershey T . Neuroinflammation in the amygdala is associated with recent depressive symptoms. Biol Psychiatry Cognit Neurosci Neuroimaging. (2023) 8:967–75. doi: 10.1016/j.bpsc.2023.04.011 37164312 PMC13386403

[47] Tsai CF Chuang CH Wang YP Lin YB Tu PC Liu PY . Differences in gut microbiota correlate with symptoms and regional brain volumes in patients with late-life depression. Front Aging Neurosci. (2022) 14:885393. doi: 10.3389/fnagi.2022.885393 35966787 PMC9365093

[48] Furness JB . The enteric nervous system and neurogastroenterology. Nat Rev Gastroenterol Hepatol. (2012) 9:286–94. doi: 10.1038/nrgastro.2012.32 22392290

[49] Sharkey KA Mawe GM . The enteric nervous system. Physiol Rev. (2023) 103:1487–564. doi: 10.69645/fwol5751 PMC9970663 36521049

[50] Bohórquez DV Samsa LA Roholt A Medicetty S Chandra R Liddle RA . An enteroendocrine cell-enteric glia connection revealed by 3D electron microscopy. PLoS One. (2014) 9:e89881. doi: 10.1371/journal.pone.0089881 24587096 PMC3935946

[51] Chudzik A Słowik T Kochalska K Pankowska A Łazorczyk A Andres-Mach M . Continuous ingestion of Lacticaseibacillus rhamnosus JB-1 during chronic stress ensures neurometabolic and behavioural stability in rats. Int J Mol Sci. (2022) 23:5173. doi: 10.3390/ijms23095173 35563564 PMC9106030

[52] Bravo JA Forsythe P Chew MV Escaravage E Savignac HM Dinan TG . Ingestion of Lactobacillus strain regulates emotional behavior and central GABA receptor expression in a mouse via the vagus nerve. Proc Natl Acad Sci USA. (2011) 108:16050–5. doi: 10.1073/pnas.1102999108 21876150 PMC3179073

[53] Perez-Burgos A Wang B Mao Y-K Mistry B McVey Neufeld KA Bienenstock J . Psychoactive bacteria Lactobacillus rhamnosus (JB-1) elicits rapid frequency facilitation in vagal afferents. (2013) 304:G211–20. doi: 10.1152/ajpgi.00128.2012 23139216

[54] Mörkl S Narrath M Schlotmann D Sallmutter MT Putz J Lang J . Multi-species probiotic supplement enhances vagal nerve function - results of a randomized controlled trial in patients with depression and healthy controls. Gut Microbes. (2025) 17:2492377. doi: 10.1080/19490976.2025.2492377 40298641 PMC12045568

[55] Najjar SA Hung LY Margolis KG . Serotonergic control of gastrointestinal development, motility, and inflammation. Compr Physiol. (2023) 13:4851–68. doi: 10.1002/j.2040-4603.2023.tb00272.x 37358510 PMC10373054

[56] Wei L Singh R Ghoshal UC . Enterochromaffin cells-gut microbiota crosstalk: Underpinning the symptoms, pathogenesis, and pharmacotherapy in disorders of gut-brain interaction. J Neurogastroenterol Motil. (2022) 28:357–75. doi: 10.5056/jnm22008 35719046 PMC9274469

[57] Hwang YK Oh JS . Interaction of the vagus nerve and serotonin in the gut-brain axis. Int J Mol Sci. (2025) 26:1160. doi: 10.3390/ijms26031160 39940928 PMC11818468

[58] Ou W Chen Y Ju Y Ma M Qin Y Bi Y . The kynurenine pathway in major depressive disorder under different disease states: A systematic review and meta-analysis. J Affect Disord. (2023) 339:624–32. doi: 10.1016/j.jad.2023.07.078 37467793

[59] Sales PMG Schrage E Coico R Pato M . Linking nervous and immune systems in psychiatric illness: A meta-analysis of the kynurenine pathway. Brain Res. (2023) 1800:148190. doi: 10.1016/j.brainres.2022.148190 36463958

[60] Yunes RA Poluektova EU Dyachkova MS Klimina KM Kovtun AS Averina OV . GABA production and structure of gadB/gadC genes in Lactobacillus and Bifidobacterium strains from human microbiota. Anaerobe. (2016) 42:197–204. doi: 10.1016/j.anaerobe.2016.10.011 27794467

[61] Bharwani A West C Champagne-Jorgensen K McVey Neufeld KA Ruberto J Kunze WA . The vagus nerve is necessary for the rapid and widespread neuronal activation in the brain following oral administration of psychoactive bacteria. Neuropharmacology. (2020) 170:108067. doi: 10.1016/j.neuropharm.2020.108067 32224131

[62] Church JS Bannish JAM Adrian LA Rojas Martinez K Henshaw A Schwartzer JJ . Serum short chain fatty acids mediate hippocampal BDNF and correlate with decreasing neuroinflammation following high pectin fiber diet in mice. Front Neurosci. (2023) 17:1134080. doi: 10.3389/fnins.2023.1134080 37123365 PMC10130583

[63] Kazemi A Noorbala AA Azam K Eskandari MH Djafarian K . Effect of probiotic and prebiotic vs placebo on psychological outcomes in patients with major depressive disorder: A randomized clinical trial. Clin Nutr. (2019) 38:522–8. doi: 10.1016/j.clnu.2018.04.010 29731182

[64] Nikolova VL Cleare AJ Young AH Stone JM . Updated review and meta-analysis of probiotics for the treatment of clinical depression: Adjunctive vs. stand-alone treatment. (2021) 10:647. doi: 10.3390/jcm10040647 PMC7915600 33567631

[65] Bercik P Verdu EF Foster JA Macri J Potter M Huang X . Chronic gastrointestinal inflammation induces anxiety-like behavior and alters central nervous system biochemistry in mice. Gastroenterology. (2010) 139:2102–2112.e2101. doi: 10.1053/j.gastro.2010.06.063 20600016

[66] Jin H Li M Jeong E Castro-Martinez F Zuker CS . A body–brain circuit that regulates body inflammatory responses. Nature. (2024) 630:695–703. doi: 10.1038/s41586-024-07469-y 38692285 PMC11186780

[67] Lei AA Phang VWX Lee YZ Kow ASF Tham CL Ho YC . Chronic stress-associated depressive disorders: The impact of HPA axis dysregulation and neuroinflammation on the hippocampus—a mini review. (2025) 26:2940. doi: 10.3390/ijms26072940 PMC11988747 40243556

[68] Cui L Li S Wang S Wu X Liu Y Yu W . Major depressive disorder: Hypothesis, mechanism, prevention and treatment. Signal Transduction Targeted Ther. (2024) 9:30. doi: 10.1038/s41392-024-01738-y 38331979 PMC10853571

[69] Dong TS Mayer E . Advances in brain-gut-microbiome interactions: A comprehensive update on signaling mechanisms, disorders, and therapeutic implications. Cell Mol Gastroenterol Hepatol. (2024) 18:1–13. doi: 10.1016/j.jcmgh.2024.01.024 38336171 PMC11126987

[70] Zong Y Zhu S Zhang S Zheng G Wiley JW Hong S . Chronic stress and intestinal permeability: Lubiprostone regulates glucocorticoid receptor-mediated changes in colon epithelial tight junction proteins, barrier function, and visceral pain in the rodent and human. Neurogastroenterol Motil. (2019) 31:e13477. doi: 10.1111/nmo.13477 30284340 PMC6347514

[71] Bertollo AG Santos CF Bagatini MD Ignácio ZM . Hypothalamus-pituitary-adrenal and gut-brain axes in biological interaction pathway of the depression. (2025) 19:2025. doi: 10.3389/fnins.2025.1541075 PMC11839829 39981404

[72] Hasegawa M Kawaguchi T Kiyohara H Teratani T Nakamoto N Mikami Y . Neural regulation of gut inflammation via autonomic nerves: Therapeutic implications for inflammatory bowel disease. Immunol Med. (2025), 1–24. doi: 10.1080/25785826.2025.2604347 41428961

[73] Lamotte G Shouman K Benarroch EE . Stress and central autonomic network. Auton Neurosci. (2021) 235:102870. doi: 10.1016/j.autneu.2021.102870 34461325

[74] Schneider KM Blank N Alvarez Y Thum K Lundgren P Litichevskiy L . The enteric nervous system relays psychological stress to intestinal inflammation. Cell. (2023) 186:2823–2838.e2820. doi: 10.1016/j.cell.2023.05.001 37236193 PMC10330875

[75] Huang Y Li H Zhu B Feng S Liu C Zhang Z . The association between gut microbiota and functional connectivity in cognitive impairment of first-episode major depressive disorder. Transl Psychiatry. (2025) 15:449. doi: 10.1038/s41398-025-03615-w 41173853 PMC12579243

[76] Kunugi H . Depression and lifestyle: Focusing on nutrition, exercise, and their possible relevance to molecular mechanisms. Psychiatry Clin Neurosci. (2023) 77:420–33. doi: 10.1111/pcn.13551 36992617 PMC11488618

[77] Bermingham KM Stensrud S Asnicar F Valdes AM Franks PW Wolf J . Exploring the relationship between social jetlag with gut microbial composition, diet and cardiometabolic health, in the ZOE PREDICT 1 cohort. Eur J Nutr. (2023) 62:3135–47. doi: 10.1007/s00394-023-03204-x 37528259 PMC10611873

[78] Liu L Nguyen SM Wang L Shi J Long J Cai Q . Associations of alcohol intake with gut microbiome: A prospective study in a predominantly low-income Black/African American population. Am J Clin Nutr. (2025) 121:134–40. doi: 10.1016/j.ajcnut.2024.11.007 39537028 PMC11747185

[79] Bui TA O'Croinin BR Dennett L Winship IR Greenshaw AJ . Pharmaco-psychiatry and gut microbiome: A systematic review of effects of psychotropic drugs for bipolar disorder. Microbiol (Reading). (2025) 171:001568. doi: 10.1099/mic.0.001568 40528728 PMC12282230

[80] Su X Tian Z Fang Y Zhou S Ma S . Effects of high-dose glucocorticoids on gut microbiota in the treatment of Graves’ ophthalmopathy. Microbiol Spectr. (2025) 13:e02467–02424. doi: 10.1128/spectrum.02467-24 40261021 PMC12131860

[81] Aasmets O Taba N Krigul KL Andreson R Estonian Biobank Research Team Org E . A hidden confounder for microbiome studies: Medications used years before sample collection. mSystems. (2025) 10:e0054125. doi: 10.1128/msystems.00541-25 40910778 PMC12542737

[82] Dilmore AH Kuplicki R McDonald D Kumar M Estaki M Youngblut N . Medication use is associated with distinct microbial features in anxiety and depression. Mol Psychiatry. (2025) 30:2545–57. doi: 10.1038/s41380-024-02857-2 39794490 PMC12092254

[83] Messaoud A Rym M Wahiba D Neffati F Najjar MF Gobbi G . Investigation of the relationship among cortisol, pro-inflammatory cytokines, and the degradation of tryptophan into kynurenine in patients with major depression and suicidal behavior. Curr Top Med Chem. (2022) 22:2119–25. doi: 10.2174/1568026621666210909160210 34503408

[84] Ogyu K Kubo K Noda Y Iwata Y Tsugawa S Omura Y . Kynurenine pathway in depression: A systematic review and meta-analysis. Neurosci Biobehav Rev. (2018) 90:16–25. doi: 10.1016/j.neubiorev.2018.03.023 29608993

[85] Schwarcz R Stone TW . The kynurenine pathway and the brain: Challenges, controversies and promises. Neuropharmacology. (2017) 112:237–47. doi: 10.1016/j.neuropharm.2016.08.003 27511838 PMC5803785

[86] Laursen MF Sakanaka M von Burg N Mörbe U Andersen D Moll JM . Bifidobacterium species associated with breastfeeding produce aromatic lactic acids in the infant gut. Nat Microbiol. (2021) 6:1367–82. doi: 10.1038/s41564-021-00970-4 34675385 PMC8556157

[87] Forton C Devries J Lou M Brundin S Cave T Anis E . Gut microbiome-derived tryptophan metabolites predict relapse in alcohol use disorder. Brain Behav Immun. (2026) 131:106161. doi: 10.1016/j.bbi.2025.106161 41192697

[88] Liu M Lu Y Xue G Han L Jia H Wang Z . Role of short-chain fatty acids in host physiology. (2024) 7:641–52. doi: 10.1002/ame2.12464 PMC11528394 38940192

[89] Ottria R Mirmajidi S Ciuffreda P . Gut microbiota-derived short-chain fatty acids in inflammatory bowel disease: Mechanistic insights into gut inflammation, barrier function, and therapeutic potential. Int J Mol Sci. (2026) 27:1095. doi: 10.3390/ijms27021095 41596739 PMC12841995

[90] Molska M Mruczyk K Cisek-Woźniak A Prokopowicz W Szydełko P Jakuszewska Z . The influence of intestinal microbiota on BDNF levels. Nutrients. (2024) 16:2891. doi: 10.3390/nu16172891 39275207 PMC11397622

[91] Mandal G Alboni S Cattane N Marizzoni M Saleri S Arslanovski N . The dietary ligands, omega-3 fatty acid endocannabinoids and short-chain fatty acids prevent cytokine-induced reduction of human hippocampal neurogenesis and alter the expression of genes involved in neuroinflammation and neuroplasticity. Mol Psychiatry. (2025) 30:5338–55. doi: 10.1038/s41380-025-03119-5 40670679 PMC12532594

[92] Schiweck C Dalile B Balliet A Aichholzer M Reinken H Erhardt F . Circulating short chain fatty acids are associated with depression severity and predict remission from major depressive disorder. Brain Behav Immun Health. (2025) 48:101070. doi: 10.1016/j.bbih.2025.101070 40761299 PMC12320157

[93] Monsalve FA Fernández-Tapia B Arriagada OC González DR Delgado-López F . Obesity and depression: A pathophysiotoxic relationship. (2025) 26:11590. doi: 10.3390/ijms262311590 PMC12692040 41373743

[94] Qiao Y Rong L Chen H Guo J Li G Wang Q . Gut microbiota, nutrients, and depression. (2025) 12:2025. doi: 10.3389/fnut.2025.1581848 PMC12568435 41170359

[95] Fleishman JS Kumar S . Bile acid metabolism and signaling in health and disease: Molecular mechanisms and therapeutic targets. Signal Transduction Targeted Ther. (2024) 9:97. doi: 10.1038/s41392-024-01811-6 38664391 PMC11045871

[96] Chen X Zhou Q He Y Wang Y Jiang Y Ren Y . TGR5 dysfunction underlies chronic social defeat stress via cAMP/PKA signaling pathway in the hippocampus. Transl Psychiatry. (2025) 15:366. doi: 10.1038/s41398-025-03599-7 41053031 PMC12501386

[97] Budoff MJ de Oliveira Otto MC Li XS Lee Y Wang M Lai HTM . Trimethylamine-N-oxide (TMAO) and risk of incident cardiovascular events in the multi ethnic study of Atherosclerosis. Sci Rep. (2025) 15:23362. doi: 10.1038/s41598-025-05903-3 40603925 PMC12222875

[98] Ge P Duan H Tao C Niu S Hu Y Duan R . TMAO promotes NLRP3 inflammasome activation of microglia aggravating neurological injury in ischemic stroke through FTO/IGF2BP2. J Inflammation Res. (2023) 16:3699–714. doi: 10.2147/jir.s399480 37663757 PMC10473438

[99] Ellis JL Karl JP Oliverio AM Fu X Soares JW Wolfe BE . Dietary vitamin K is remodeled by gut microbiota and influences community composition. Gut Microbes. (2021) 13:1–16. doi: 10.1080/19490976.2021.1887721 33651646 PMC7928036

[100] Sloof WG Pei R Mcdonald SA Fife JL Shen L Boatemaa L . Repeated crack healing in MAX-phase ceramics revealed by 4D in situ synchrotron X-ray tomographic microscopy. Sci Rep. (2016) 6:23040. doi: 10.1038/srep23040 26972608 PMC4789783

[101] Jacka FN O'Neil A Opie R Itsiopoulos C Cotton S Mohebbi M . A randomised controlled trial of dietary improvement for adults with major depression (the 'SMILES' trial). BMC Med. (2017) 15:23. doi: 10.1186/s12916-017-0791-y 28137247 PMC5282719

[102] Paris T Daly RM Abbott G Sood S Freer CL Ryan MC . Diet overall and hypocaloric diets are associated with improvements in depression but not anxiety in people with metabolic conditions: A systematic review and meta-analysis. Adv Nutr. (2024) 15:100169. doi: 10.1016/j.advnut.2024.100169 38184198 PMC10847486

[103] Wang R Cai Y Lu W Zhang R Shao R Yau SY . Exercise effect on the gut microbiota in young adolescents with subthreshold depression: A randomized psychoeducation-controlled trial. Psychiatry Res. (2023) 319:115005. doi: 10.1016/j.psychres.2022.115005 36565548

[104] Hamada H Oyanagi E Watanabe C Aoki T Kawashima M Yajima H . Physical activity is associated with reduced mild depression and altered gut microbiota in Japanese adult women. Cureus. (2025) 17:e90104. doi: 10.7759/cureus.90104 40955250 PMC12433704

[105] Yao M Qu Y Zheng Y Guo H . The effect of exercise on depression and gut microbiota: Possible mechanisms. Brain Res Bull. (2025) 220:111130. doi: 10.1016/j.brainresbull.2024.111130 39557221

[106] Shan W Zang W Zuo Z . Sleep deprivation disrupts diurnal rhythmicity of gut microbiota and blood inflammatory cytokines in mice. PLoS One. (2025) 20:e0335754. doi: 10.1371/journal.pone.0335754 41202022 PMC12594415

[107] Salminen S Collado MC Endo A Hill C Lebeer S Quigley EMM . The International Scientific Association of Probiotics and Prebiotics (ISAPP) consensus statement on the definition and scope of postbiotics. Nat Rev Gastroenterol Hepatol. (2021) 18:649–67. doi: 10.1038/s41575-021-00440-6 33948025 PMC8387231

[108] Zhang J Zhu L Meng Q Wang Z Zhu H . The efficacy of probiotics, prebiotics, and synbiotics on anxiety, depression, and sleep: a systematic review and meta-analysis of randomized controlled trials. BMC Psychiatry. (2025) 25:1199. doi: 10.1186/s12888-025-07644-z 41310510 PMC12751681

[109] Nikolova VL Cleare AJ Young AH Stone JM . Acceptability, tolerability, and estimates of putative treatment effects of probiotics as adjunctive treatment in patients with depression: A randomized clinical trial. JAMA Psychiatry. (2023) 80:842–7. doi: 10.1001/jamapsychiatry.2023.1817 37314797 PMC10267847

[110] Zhang Q Chen B Zhang J Dong J Ma J Zhang Y . Effect of prebiotics, probiotics, synbiotics on depression: Results from a meta-analysis. BMC Psychiatry. (2023) 23:477. doi: 10.1186/s12888-023-04963-x 37386630 PMC10308754

[111] Yoo S Jung S-C Kwak K Kim JS . The role of prebiotics in modulating gut microbiota: Implications for human health. (2024) 25:4834. doi: 10.3390/ijms25094834 PMC11084426 38732060

[112] Baek J Lee S Lee J Park J Choi E Kang SS . Utilization of probiotic-derived extracellular vesicles as postbiotics and their role in mental health therapeutics. Food Sci Anim Resour. (2024) 44:1252–65. doi: 10.5851/kosfa.2024.e92 39554832 PMC11564138

[113] Misera A Liśkiewicz P Łoniewski I Skonieczna-Żydecka K Samochowiec J . Effect of psychobiotics on psychometric tests and inflammatory markers in major depressive disorder: Meta-analysis of randomized controlled trials with meta-regression. Pharm (Basel). (2021) 14:952. doi: 10.3390/ph14100952 34681176 PMC8541446

[114] Rahmannia M Poudineh M Mirzaei R Aalipour MA Shahidi Bonjar AH Goudarzi M . Strain-specific effects of probiotics on depression and anxiety: A meta-analysis. Gut Pathog. (2024) 16:46. doi: 10.1186/s13099-024-00634-8 39245752 PMC11382490

[115] Doll JPK Vázquez-Castellanos JF Schaub AC Schweinfurth N Kettelhack C Schneider E . Fecal microbiota transplantation (FMT) as an adjunctive therapy for depression-case report. Front Psychiatry. (2022) 13:815422. doi: 10.3389/fpsyt.2022.815422 35250668 PMC8891755

[116] Wang L Zhang S Liu Y Li D Tian G Li X . A study on the efficacy and safety of fecal microbiota transplantation as an adjunctive therapy for treating depressive episodes. Sci Rep. (2026) 16:13417. doi: 10.1038/s41598-026-41801-y 41826399 PMC13111675

[117] Moshfeghinia R Nemati H Ebrahimi A Shekouh D Karami S Eraghi MM . The impact of probiotics, prebiotics, and synbiotics on depression and anxiety symptoms of patients with depression: A systematic review and meta-analysis. J Psychiatr Res. (2025) 188:104–16. doi: 10.1016/j.jpsychires.2025.05.053 40440772

[118] Wessa C Simon MS De Picker L . Current evidence on immune-driven depression. Curr Opin Psychiatry. (2026) 39:8–18. doi: 10.1097/yco.0000000000001047 41217347 PMC12672047

[119] Lee SM Dong TS Krause-Sorio B Siddarth P Milillo MM Lagishetty V . The intestinal microbiota as a predictor for antidepressant treatment outcome in geriatric depression: A prospective pilot study. Int Psychogeriatrics. (2022) 34:33–45. doi: 10.1017/s1041610221000120 33757609

[120] Merlo G Bachtel G Sugden SG . Gut microbiota, nutrition, and mental health. (2024) 11:2024. doi: 10.3389/fnut.2024.1337889 PMC10884323 38406183

[121] Zeng L Zhang S Liu R Wang L Tan Y . The microbiota-gut-brain axis in depression: Mechanisms, microbiota-targeted interventions, and translational challenges. Int J Microbiol. (2025) 2025:6750078. doi: 10.1155/ijm/6750078 41476898 PMC12752827

[122] Zol N Hasain Z Shakrin N Jati AKK Chong CW Loganathan AL . Methodological insights into gut microbiome profiling: A comprehensive review with emphasis on bipolar disorder and major depressive disorder. Malaysian J Med Health Sci. (2025) 21:1–16. doi: 10.47836/mjmhs.v21.i6.1359

[123] Broeckling CD Beger RD Cheng LL Cumeras R Cuthbertson DJ Dasari S . Current practices in LC-MS untargeted metabolomics: A scoping review on the use of pooled quality control samples. Anal Chem. (2023) 95:18645–54. doi: 10.1021/acs.analchem.3c02924 38055671 PMC10753522

[124] Zeki ÖC Eylem CC Reçber T Kır S Nemutlu E . Integration of GC–MS and LC–MS for untargeted metabolomics profiling. J Pharm BioMed Anal. (2020) 190:113509. doi: 10.1016/j.jpba.2020.113509 32814263

[125] Penninx BWJH Lamers F Jansen R Berk M Khandaker GM De Picker L . Immuno-metabolic depression: From concept to implementation. Lancet Regional Health – Europe. (2025) 48:101166. doi: 10.1016/j.lanepe.2024.101166 39801616 PMC11721223

[126] Wang Y Zhou J Ye J Sun Z He Y Zhao Y . Multi-omics reveal microbial determinants impacting the treatment outcome of antidepressants in major depressive disorder. Microbiome. (2023) 11:195. doi: 10.1186/s40168-023-01635-6 37641148 PMC10464022

